# Supplementary material for: Site-specific free energy surface parameters from single-molecule fluorescence measurements of exciton-coupled (iCy3)2 dimer probes positioned at DNA replication fork junctions
Source: Nucleic Acids Res. 2025 Feb 5;53(3):gkaf047. doi: 10.1093/nar/gkaf047 (PMC11795206; doi:10.1093/nar/gkaf047)
Supplement: gkaf047_Supplemental_Files [file gkaf047_supplemental_files.zip › Maurer-paper-SI.v12.pdf]

Supporting Information: Site-specific free energy surface parameters from single-molecule fluorescence measurements of exciton-coupled $(iCy3)_2$ dimer probes positioned at DNA replication fork junctions

Jack Maurer,[1,2,3] Claire S. Albrecht,[1,2,4] Peter H. von Hippel[2,3] and Andrew H. Marcus[1,2,3,*]

[1.] Center for Optical, Molecular and Quantum Science, University of Oregon, Eugene, Oregon 97403

[2.] Institute of Molecular Biology, University of Oregon, Eugene, Oregon 97403

[3.] Department of Chemistry and Biochemistry, University of Oregon, Eugene, Oregon 97403

[4.] Department of Physics, University of Oregon, Eugene, Oregon 97403

* Corresponding author email: ahmarcus@uoregon.edu

Table S1. Optimized parameters for the Gaussian components of the PS-SMF probability distribution functions (PDFs).

Sample	A_1	$\bar{v}_1$	σ_1	A_2	$\bar{v}_2$	σ_2	A_3	$\bar{v}_3$	σ_3	A_4	$\bar{v}_4$	σ_4	A_5	$\bar{v}_5$	σ_5
+1, 300 mM NaCl	0.08	0.003	0.037	0.59	0.11	0.049	4.84	0.15	0.075	0.02	0.32	0.072	0.12	0.39	0.054
+1, 100 mM NaCl	0.15	0.06	0.059	6.11	0.11	0.053	1.02	0.14	0.053	0.18	0.35	0.048	-	-	-
+1, 20mM NaCl	1.79	0.06	0.055	6.07	0.11	0.048	0.32	0.17	0.032	0.02	0.30	0.053	-	-	-
+1, 20mM NaCl, 0 mM $MgCl_2$	0.12	0.0001	0.019	5.31	0.12	0.062	1.01	0.14	0.060	0.19	0.37	0.028	-	-	-
-1, 100mM NaCl	0.22	0.0001	0.045	2.29	0.10	0.037	5.50	0.12	0.054	0.002	0.30	0.049	-	-	-
-2, 300mM NaCl	0.97	0.04	0.039	6.81	0.12	0.051	0.37	0.15	0.031	0.02	0.30	0.039	-	-	-
-2, 100mM NaCl	0.78	0.04	0.027	3.46	0.09	0.023	4.48	0.13	0.063	0.20	0.30	0.063	-	-	-
-2, 20mM NaCl	0.40	0.0001	0.038	6.71	0.12	0.054	0.39	0.17	0.044	0.07	0.30	0.02	-	-	-
-2, 20mM NaCl, 0 mM $MgCl_2$	1.43	0.06	0.034	6.32	0.11	0.037	1.31	0.19	0.045	1.09	0.24	0.045	0.01	0.32	0.046

Parameters defined in Eq. (5). Error bars for the mean visibilities are < 1% of reported values (see Statistical Uncertainty Analysis below). PDFs were constructed using the integration period T_w = 10 ms. All samples prepared in 10 mM Tris at pH 8.0 and 6 mM $MgCl_2$, unless otherwise specified.

Table S2. Optimized values of the equilibrium probabilities and the visibility fluctuations.

Sample	p_1^{eq}	p_2^{eq}	p_3^{eq}	p_4^{eq}	p_5^{eq}	$\delta\bar{v}_1$	$\delta\bar{v}_2$	$\delta\bar{v}_3$	$\delta\bar{v}_4$	$\delta\bar{v}_5$	$\bar{v}$
+1, 300 mM NaCl	0.01	0.07	0.91	0.003	0.02	-0.15	-0.04	0.00	0.17	0.24	0.15
+1, 100 mM NaCl	0.02	0.82	0.14	0.02	-	-0.06	-0.01	0.02	0.23	-	0.12
+1, 20mM NaCl	0.25	0.74	0.02	0.002	-	-0.04	0.01	0.07	0.20	-	0.10
+1, 20mM NaCl, 0 mM $MgCl_2$	0.01	0.82	0.15	0.01	-	-0.12	0.00	0.02	0.25	-	0.12
-1, 100mM NaCl	0.02	0.22	0.76	0.0003	-	-0.11	-0.01	0.01	0.19	-	0.11
-2, 300mM NaCl	0.10	0.88	0.03	0.001	-	-0.07	0.01	0.04	0.19	-	0.11
-2, 100mM NaCl	0.05	0.20	0.71	0.03	-	-0.08	-0.03	0.01	0.18	-	0.12
-2, 20mM NaCl	0.04	0.91	0.04	0.003	-	-0.12	0.00	0.05	0.18	-	0.12
-2, 20mM NaCl, 0 mM $MgCl_2$	0.12	0.60	0.15	0.12	0.001	-0.07	-0.02	0.06	0.11	0.19	0.13

Equilibrium probabilities are calculated using the parameters listed in Table S1 and $p_i^{eq} = A_i\sigma_i\sqrt{2\pi}$. Visibility fluctuations are calculated using the mean visibilities listed in Table S1 and $\delta\bar{v}_i = \bar{v}_i - \bar{v}$, where $\bar{v} = \sum_{i=1}^{4} p_i^{eq}\,\bar{v}_i$.

Table S3. Decomposition of largest pathway terms for the two-point TCFs [$\bar{C}^{(2)}$function, Eq. (6) of main text] for τ = 250 µs.

+1, 300 mM NaCl, 6 mM $MgCl_2$									
Positive terms					Negative terms				
i	j	$\delta\bar{v}_i\delta\bar{v}_j$	w_{ij}	$w_{ij}\delta\bar{v}_i\delta\bar{v}_j$	i	j	$\delta\bar{v}_i\delta\bar{v}_j$	w_{ij}	$w_{ij}\delta\bar{v}_i\delta\bar{v}_j$
5	5	5.70×10^{-2}	1.40×10^{-2}	7.98×10^{-4}	1	5	-3.58×10^{-2}	1.84×10^{-3}	-6.60×10^{-5}
1	1	2.24×10^{-2}	5.94×10^{-3}	1.33×10^{-4}	5	1	-3.58×10^{-2}	1.84×10^{-3}	-6.60×10^{-5}
4	4	2.82×10^{-2}	3.96×10^{-3}	1.12×10^{-4}	3	5	-6.45×10^{-4}	3.79×10^{-4}	-2.44×10^{-7}
2	2	1.47×10^{-3}	7.31×10^{-2}	1.07×10^{-4}	5	3	-6.45×10^{-4}	3.79×10^{-4}	-2.44×10^{-7}
3	3	7.29×10^{-6}	9.10×10^{-1}	6.64×10^{-6}	2	5	-9.15×10^{-3}	4.35×10^{-6}	-3.98×10^{-8}
2	1	5.74×10^{-3}	2.98×10^{-5}	1.71×10^{-7}	5	2	-9.15×10^{-3}	3.75×10^{-6}	-3.43×10^{-8}
1	2	5.74×10^{-3}	2.57×10^{-5}	1.47×10^{-7}	4	3	-4.54×10^{-4}	2.72×10^{-6}	-1.24×10^{-9}
3	1	4.04×10^{-4}	2.51×10^{-5}	1.02×10^{-8}	3	4	-4.54×10^{-4}	1.92×10^{-6}	-8.73×10^{-10}

+1, 100 mM NaCl, 6 mM $MgCl_2$									
Positive terms					Negative terms				
i	j	$\delta\bar{v}_i\delta\bar{v}_j$	w_{ij}	$w_{ij}\delta\bar{v}_i\delta\bar{v}_j$	i	j	$\delta\bar{v}_i\delta\bar{v}_j$	w_{ij}	$w_{ij}\delta\bar{v}_i\delta\bar{v}_j$
4	4	5.28×10^{-2}	9.07×10^{-3}	4.79×10^{-4}	1	4	-1.44×10^{-2}	7.06×10^{-4}	-1.02×10^{-5}
1	1	3.91×10^{-3}	2.08×10^{-2}	8.12×10^{-5}	4	1	-1.44×10^{-2}	7.06×10^{-4}	-1.01×10^{-5}
4	3	4.25×10^{-3}	1.15×10^{-2}	4.90×10^{-5}	1	3	-1.16×10^{-3}	3.42×10^{-4}	-3.96×10^{-7}
3	4	4.25×10^{-3}	1.15×10^{-2}	4.88×10^{-5}	3	1	-1.16×10^{-3}	3.41×10^{-4}	-3.95×10^{-7}
2	2	5.62×10^{-5}	8.19×10^{-1}	4.60×10^{-5}	2	4	-1.72×10^{-3}	7.67×10^{-5}	-1.32×10^{-7}
3	3	3.43×10^{-4}	1.26×10^{-1}	4.32×10^{-5}	4	2	-1.72×10^{-3}	4.01×10^{-5}	-6.91×10^{-8}
2	1	4.69×10^{-4}	2.21×10^{-6}	1.04×10^{-9}	3	2	-1.39×10^{-4}	7.74×10^{-5}	-1.08×10^{-8}
1	2	4.69×10^{-4}	1.12×10^{-6}	5.28×10^{-10}	2	3	-1.39×10^{-4}	3.96×10^{-5}	-5.50×10^{-9}

+1, 20 mM NaCl, 6 mM $MgCl_2$									
Positive terms					Negative terms				
i	j	$\delta\bar{v}_i\delta\bar{v}_j$	w_{ij}	$w_{ij}\delta\bar{v}_i\delta\bar{v}_j$	i	j	$\delta\bar{v}_i\delta\bar{v}_j$	w_{ij}	$w_{ij}\delta\bar{v}_i\delta\bar{v}_j$
1	1	1.76×10^{-3}	1.64×10^{-3}	2.89×10^{-4}	1	2	-3.38×10^{-4}	8.48×10^{-2}	-2.87×10^{-5}
4	4	3.53×10^{-2}	2.66×10^{-3}	9.42×10^{-5}	2	1	-3.38×10^{-4}	8.48×10^{-2}	-2.87×10^{-5}
3	3	3.52×10^{-3}	2.56×10^{-2}	9.02×10^{-5}	4	1	-7.89×10^{-3}	3.84×10^{-5}	-3.03×10^{-7}
2	2	6.50×10^{-5}	6.60×10^{-1}	4.28×10^{-5}	1	4	-7.89×10^{-3}	3.84×10^{-4}	-3.03×10^{-7}
4	2	1.51×10^{-3}	8.88×10^{-6}	1.34×10^{-8}	3	1	-2.49×10^{-3}	6.24×10^{-7}	-1.56×10^{-9}
2	4	1.51×10^{-3}	8.88×10^{-6}	1.34×10^{-8}	1	3	-2.49×10^{-3}	9.72×10^{-8}	-2.42×10^{-10}
3	2	4.78×10^{-5}	9.33×10^{-6}	4.47×10^{-9}					
2	3	4.78×10^{-4}	1.45×10^{-6}	6.95×10^{-10}					

+1, 20 mM NaCl, 0 mM $MgCl_2$									
Positive terms					Negative terms				
i	j	$\delta\bar{v}_i\delta\bar{v}_j$	w_{ij}	$w_{ij}\delta\bar{v}_i\delta\bar{v}_j$	i	j	$\delta\bar{v}_i\delta\bar{v}_j$	w_{ij}	$w_{ij}\delta\bar{v}_i\delta\bar{v}_j$
4	4	5.80×10^{-2}	7.17×10^{-3}	4.16×10^{-4}	1	3	-1.75×10^{-3}	3.03×10^{-4}	-5.30×10^{-7}
1	1	1.64×10^{-2}	5.61×10^{-3}	9.27×10^{-5}	3	1	-1.75×10^{-3}	3.03×10^{-4}	-5.30×10^{-7}
3	3	1.85×10^{-4}	1.48×10^{-1}	2.74×10^{-5}	2	4	-1.36×10^{-3}	2.09×10^{-4}	-2.83×10^{-7}
2	2	3.18×10^{-5}	8.25×10^{-1}	2.62×10^{-5}	4	1	-3.10×10^{-2}	7.43×10^{-6}	-2.30×10^{-7}
4	3	3.28×10^{-3}	6.54×10^{-3}	2.14×10^{-5}	1	4	-3.10×10^{-2}	7.21×10^{-6}	-2.23×10^{-7}
3	4	3.28×10^{-3}	6.35×10^{-3}	2.08×10^{-5}	3	2	-7.67×10^{-5}	3.49×10^{-4}	-2.68×10^{-8}
1	2	7.24×10^{-4}	3.55×10^{-7}	2.57×10^{-10}	4	2	-1.35×10^{-3}	1.53×10^{-5}	-2.07×10^{-8}
2	1	7.24×10^{-4}	1.36×10^{-7}	9.86×10^{-11}	2	3	-7.67×10^{-5}	1.56×10^{-4}	-1.19×10^{-8}

-1, 100 mM NaCl, 6 mM $MgCl_2$									
Positive terms					Negative terms				
i	j	$\delta\bar{v}_i\delta\bar{v}_j$	w_{ij}	$w_{ij}\delta\bar{v}_i\delta\bar{v}_j$	i	j	$\delta\bar{v}_i\delta\bar{v}_j$	w_{ij}	$w_{ij}\delta\bar{v}_i\delta\bar{v}_j$
1	1	1.31×10^{-2}	2.06×10^{-2}	2.70×10^{-4}	3	1	-8.88×10^{-4}	4.08×10^{-3}	-3.63×10^{-6}
3	3	6.02×10^{-5}	7.52×10^{-1}	4.53×10^{-5}	1	3	-8.88×10^{-4}	3.97×10^{-3}	-3.53×10^{-6}
2	2	1.85×10^{-4}	2.16×10^{-1}	4.00×10^{-5}	2	3	-1.06×10^{-4}	2.36×10^{-4}	-2.49×10^{-8}
4	4	3.44×10^{-2}	2.75×10^{-4}	9.47×10^{-6}	4	1	-2.12×10^{-2}	7.44×10^{-7}	-1.58×10^{-8}
1	2	1.56×10^{-3}	1.35×10^{-3}	2.10×10^{-6}	1	4	-2.12×10^{-2}	7.44×10^{-7}	-1.58×10^{-8}
2	1	1.56×10^{-3}	1.24×10^{-3}	1.93×10^{-6}	3	2	-1.06×10^{-4}	1.26×10^{-4}	-1.33×10^{-8}
3	4	1.44×10^{-3}	6.83×10^{-8}	9.84×10^{-11}	4	2	-2.52×10^{-3}	2.26×10^{-8}	-5.70×10^{-11}
4	3	1.44×10^{-3}	6.65×10^{-8}	9.57×10^{-11}	2	4	-2.52×10^{-3}	2.07×10^{-8}	-5.24×10^{-11}

-2, 300 mM NaCl, 6 mM $MgCl_2$									
Positive terms					Negative terms				
i	j	$\delta\bar{v}_i\delta\bar{v}_j$	w_{ij}	$w_{ij}\delta\bar{v}_i\delta\bar{v}_j$	i	j	$\delta\bar{v}_i\delta\bar{v}_j$	w_{ij}	$w_{ij}\delta\bar{v}_i\delta\bar{v}_j$
1	1	5.52×10^{-3}	5.27×10^{-2}	2.91×10^{-4}	1	2	-3.89×10^{-4}	4.27×10^{-2}	-1.66×10^{-5}
4	4	3.44×10^{-2}	1.88×10^{-3}	6.46×10^{-5}	2	1	-3.89×10^{-4}	4.27×10^{-2}	-1.66×10^{-5}
3	3	1.74×10^{-3}	2.88×10^{-2}	5.02×10^{-5}	4	1	-1.38×10^{-2}	3.28×10^{-5}	-4.52×10^{-7}
2	2	2.74×10^{-5}	8.39×10^{-1}	2.30×10^{-5}	1	4	-1.38×10^{-2}	3.28×10^{-5}	-4.52×10^{-7}
4	2	9.71×10^{-4}	1.09×10^{-5}	1.06×10^{-8}	3	1	-3.10×10^{-3}	3.74×10^{-7}	-1.16×10^{-9}
2	4	9.71×10^{-4}	1.09×10^{-5}	1.06×10^{-8}	1	3	-3.10×10^{-3}	2.72×10^{-7}	-8.46×10^{-10}
3	2	2.18×10^{-4}	1.35×10^{-5}	2.95×10^{-9}					
2	3	2.18×10^{-4}	9.84×10^{-6}	2.15×10^{-9}					

-2, 100 mM NaCl, 6 mM $MgCl_2$									
Positive terms					Negative terms				
i	j	$\delta\bar{v}_i\delta\bar{v}_j$	w_{ij}	$w_{ij}\delta\bar{v}_i\delta\bar{v}_j$	i	j	$\delta\bar{v}_i\delta\bar{v}_j$	w_{ij}	$w_{ij}\delta\bar{v}_i\delta\bar{v}_j$
4	4	3.02×10^{-2}	1.98×10^{-2}	5.98×10^{-4}	4	1	-1.33×10^{-2}	1.15×10^{-2}	-1.54×10^{-5}
1	1	5.86×10^{-3}	3.74×10^{-2}	2.19×10^{-4}	1	4	-1.33×10^{-2}	1.15×10^{-2}	-1.54×10^{-5}
2	2	9.22×10^{-4}	1.97×10^{-1}	1.82×10^{-4}	2	4	-5.28×10^{-3}	7.63×10^{-4}	-4.03×10^{-6}
3	3	4.41×10^{-5}	7.10×10^{-1}	3.13×10^{-5}	4	2	-5.28×10^{-3}	7.62×10^{-4}	-4.02×10^{-6}
1	2	2.32×10^{-3}	4.95×10^{-3}	1.15×10^{-5}	2	3	-2.02×10^{-4}	4.57×10^{-4}	-9.22×10^{-8}
2	1	2.32×10^{-3}	4.95×10^{-3}	1.15×10^{-5}	3	2	-2.02×10^{-4}	4.57×10^{-4}	-9.22×10^{-8}
3	4	1.15×10^{-3}	6.32×10^{-7}	7.30×10^{-10}	1	3	-5.08×10^{-4}	6.04×10^{-6}	-3.07×10^{-9}
4	3	1.15×10^{-3}	6.30×10^{-7}	7.28×10^{-10}	3	1	-5.08×10^{-4}	6.04×10^{-6}	-3.07×10^{-9}

-2, 20 mM NaCl, 6 mM $MgCl_2$									
Positive terms					Negative terms				
i	j	$\delta\bar{v}_i\delta\bar{v}_j$	w_{ij}	$w_{ij}\delta\bar{v}_i\delta\bar{v}_j$	i	j	$\delta\bar{v}_i\delta\bar{v}_j$	w_{ij}	$w_{ij}\delta\bar{v}_i\delta\bar{v}_j$
1	1	1.36×10^{-2}	2.51×10^{-2}	3.40×10^{-4}	1	2	-1.95×10^{-4}	1.41×10^{-2}	-2.75×10^{-6}
3	3	2.62×10^{-3}	4.38×10^{-2}	1.15×10^{-4}	2	1	-1.95×10^{-4}	1.41×10^{-2}	-2.74×10^{-6}
4	4	3.35×10^{-2}	3.36×10^{-3}	1.12×10^{-4}	4	1	-2.13×10^{-3}	4.36×10^{-5}	-9.29×10^{-7}
2	2	2.80×10^{-6}	9.01×10^{-1}	2.52×10^{-6}	1	4	-2.13×10^{-3}	4.36×10^{-5}	-9.29×10^{-7}
4	2	3.06×10^{-4}	1.05×10^{-5}	3.22×10^{-9}	3	1	-5.96×10^{-3}	1.79×10^{-5}	-1.07×10^{-7}
2	4	3.06×10^{-4}	1.05×10^{-5}	3.22×10^{-9}	1	3	-5.96×10^{-3}	3.76×10^{-7}	-2.24×10^{-9}
2	3	8.56×10^{-5}	2.68×10^{-5}	2.30×10^{-9}					
3	2	8.56×10^{-5}	1.02×10^{-5}	8.70×10^{-10}					

-2, 20 mM NaCl, 0 mM $MgCl_2$									
Positive terms					Negative terms				
i	j	$\delta\bar{v}_i\delta\bar{v}_j$	w_{ij}	$w_{ij}\delta\bar{v}_i\delta\bar{v}_j$	i	j	$\delta\bar{v}_i\delta\bar{v}_j$	w_{ij}	$w_{ij}\delta\bar{v}_i\delta\bar{v}_j$
4	4	1.24×10^{-2}	6.11×10^{-2}	7.61×10^{-4}	4	1	-8.86×10^{-3}	6.11×10^{-2}	-5.41×10^{-4}
1	1	6.30×10^{-3}	6.11×10^{-2}	3.85×10^{-4}	1	4	-8.86×10^{-3}	6.11×10^{-2}	-5.41×10^{-4}
3	3	2.79×10^{-3}	1.20×10^{-1}	3.35×10^{-4}	2	3	-1.06×10^{-3}	2.81×10^{-2}	-2.98×10^{-5}
2	2	4.05×10^{-4}	5.73×10^{-1}	2.32×10^{-4}	3	2	-1.06×10^{-3}	2.81×10^{-2}	-2.98×10^{-5}
5	5	3.29×10^{-2}	5.90×10^{-4}	1.94×10^{-5}	1	3	-4.19×10^{-3}	1.32×10^{-3}	-5.53×10^{-6}
3	4	5.89×10^{-3}	1.32×10^{-3}	7.76×10^{-6}	3	1	-4.19×10^{-3}	1.32×10^{-3}	-5.53×10^{-6}
4	3	5.89×10^{-3}	1.32×10^{-3}	7.76×10^{-6}	2	4	-2.24×10^{-3}	1.44×10^{-4}	-3.23×10^{-7}
1	2	$1. 60 \times 10^{-3}$	1.44×10^{-4}	2.30×10^{-7}	4	2	-2.24×10^{-3}	1.44×10^{-4}	-3.23×10^{-7}

Table S4. Decomposition of largest pathway terms for the three-point TCFs [$\bar{C}^{(3)}$function, Eq. (7) of main text] for $\tau_1 = \tau_2 = 10$ ms.

+1, 300 mM NaCl, 6 mM $MgCl_2$											
Positive terms						Negative terms					
i	j	k	$\delta\bar{v}_i\delta\bar{v}_j\delta\bar{v}_k$	w_{ijk}	$w_{ijk}\delta\bar{v}_i\delta\bar{v}_j\delta\bar{v}_k$	i	j	k	$\delta\bar{v}_i\delta\bar{v}_j\delta\bar{v}_k$	w_{ijk}	$w_{ijk}\delta\bar{v}_i\delta\bar{v}_j\delta\bar{v}_k$
5	5	5	1.36×10^{-2}	1.66×10^{-3}	2.66×10^{-5}	5	1	5	-8.54×10^{-3}	8.86×10^{-4}	-7.56×10^{-6}
4	4	4	4.73×10^{-3}	3.78×10^{-3}	1.79×10^{-5}	1	5	5	-8.54×10^{-3}	8.44×10^{-4}	-7.21×10^{-6}
1	1	5	5.36×10^{-3}	4.49×10^{-4}	2.40×10^{-6}	5	5	1	-8.54×10^{-3}	8.44×10^{-4}	-7.21×10^{-6}
5	1	1	5.36×10^{-3}	4.49×10^{-4}	2.40×10^{-6}	2	2	2	-5.64×10^{-5}	7.06×10^{-2}	-3.98×10^{-6}
1	5	1	5.36×10^{-3}	4.28×10^{-4}	2.29×10^{-6}	1	1	1	-3.36×10^{-3}	2.28×10^{-4}	-7.65×10^{-7}
2	2	5	3.51×10^{-4}	6.03×10^{-4}	2.12×10^{-7}	2	5	5	-2.19×10^{-3}	1.97×10^{-4}	-4.30×10^{-7}
5	2	2	3.51×10^{-4}	5.18×10^{-4}	1.82×10^{-7}	3	5	5	-1.56×10^{-4}	2.52×10^{-3}	-3.93×10^{-7}
2	1	5	1.37×10^{-3}	1.30×10^{-4}	1.78×10^{-7}	5	5	3	-1.56×10^{-4}	2.52×10^{-3}	-3.93×10^{-7}

+1, 100 mM NaCl, 6 mM $MgCl_2$											
Positive terms						Negative terms					
i	j	k	$\delta\bar{v}_i\delta\bar{v}_j\delta\bar{v}_k$	w_{ijk}	$w_{ijk}\delta\bar{v}_i\delta\bar{v}_j\delta\bar{v}_k$	i	j	k	$\delta\bar{v}_i\delta\bar{v}_j\delta\bar{v}_k$	w_{ijk}	$w_{ijk}\delta\bar{v}_i\delta\bar{v}_j\delta\bar{v}_k$
4	4	4	1.21×10^{-2}	2.80×10^{-4}	3.40×10^{-6}	1	4	4	-3.30×10^{-3}	2.68×10^{-4}	-8.83×10^{-7}
4	3	4	9.77×10^{-4}	1.84×10^{-3}	1.80×10^{-6}	4	4	1	-3.30×10^{-3}	2.68×10^{-4}	-8.83×10^{-7}
4	4	3	9.77×10^{-4}	1.83×10^{-3}	1.79×10^{-6}	4	1	4	-3.30×10^{-3}	2.50×10^{-4}	-8.24×10^{-7}
3	4	4	9.77×10^{-4}	1.82×10^{-3}	1.78×10^{-6}	1	4	3	-2.66×10^{-4}	1.74×10^{-3}	-4.64×10^{-7}
4	3	3	7.88×10^{-5}	1.20×10^{-2}	9.47×10^{-7}	3	4	1	-2.66×10^{-4}	1.74×10^{-3}	-4.63×10^{-7}
3	3	4	7.88×10^{-5}	1.20×10^{-2}	9.44×10^{-7}	1	3	4	-2.66×10^{-4}	1.66×10^{-3}	-4.41×10^{-7}
3	4	3	7.88×10^{-5}	1.19×10^{-2}	9.36×10^{-7}	4	3	1	-2.66×10^{-4}	1.66×10^{-3}	-4.41×10^{-7}
3	3	3	6.34×10^{-6}	7.84×10^{-2}	4.898×10^{-7}	4	1	3	-2.66×10^{-4}	1.54×10^{-3}	-4.09×10^{-7}

+1, 20 mM NaCl, 6 mM $MgCl_2$											
Positive terms						Negative terms					
i	j	k	$\delta\bar{v}_i\delta\bar{v}_j\delta\bar{v}_k$	w_{ijk}	$w_{ijk}\delta\bar{v}_i\delta\bar{v}_j\delta\bar{v}_k$	i	j	k	$\delta\bar{v}_i\delta\bar{v}_j\delta\bar{v}_k$	w_{ijk}	$w_{ijk}\delta\bar{v}_i\delta\bar{v}_j\delta\bar{v}_k$
3	3	3	2.09×10^{-4}	2.51×10^{-2}	5.24×10^{-6}	1	1	1	-7.41×10^{-5}	1.56×10^{-2}	-1.16×10^{-6}
4	4	4	6.64×10^{-3}	6.65×10^{-4}	4.41×10^{-6}	1	2	2	-2.69×10^{-6}	1.39×10^{-1}	-3.75×10^{-7}
1	2	1	1.41×10^{-5}	4.67×10^{-2}	6.59×10^{-7}	2	2	1	-2.69×10^{-6}	1.39×10^{-1}	-3.75×10^{-7}
1	1	2	1.41×10^{-5}	4.67×10^{-2}	6.59×10^{-7}	2	1	2	-2.69×10^{-6}	1.39×10^{-1}	-3.75×10^{-7}
2	1	1	1.41×10^{-5}	4.67×10^{-2}	6.59×10^{-7}	4	4	1	-1.48×10^{-3}	1.85×10^{-4}	-2.75×10^{-7}
2	2	2	5.12×10^{-7}	4.16×10^{-1}	2.13×10^{-7}	1	4	4	-1.48×10^{-3}	1.85×10^{-4}	-2.75×10^{-7}
4	4	2	2.83×10^{-4}	4.93×10^{-4}	1.39×10^{-7}	4	1	2	-6.32×10^{-5}	2.79×10^{-4}	-1.76×10^{-8}
2	4	4	2.83×10^{-4}	4.93×10^{-4}	1.39×10^{-7}	2	1	4	-6.32×10^{-5}	2.79×10^{-4}	-1.76×10^{-8}

+1, 20 mM NaCl, 0 mM $MgCl_2$											
Positive terms						Negative terms					
i	j	k	$\delta\bar{v}_i\delta\bar{v}_j\delta\bar{v}_k$	w_{ijk}	$w_{ijk}\delta\bar{v}_i\delta\bar{v}_j\delta\bar{v}_k$	i	j	k	$\delta\bar{v}_i\delta\bar{v}_j\delta\bar{v}_k$	w_{ijk}	$w_{ijk}\delta\bar{v}_i\delta\bar{v}_j\delta\bar{v}_k$
4	4	4	1.40×10^{-2}	6.93×10^{-5}	9.69×10^{-7}	1	1	1	-2.12×10^{-3}	1.11×10^{-4}	-2.36×10^{-7}
4	4	3	7.90×10^{-4}	8.02×10^{-4}	6.34×10^{-7}	4	4	1	-7.46×10^{-3}	2.78×10^{-5}	-2.07×10^{-7}
4	3	4	7.90×10^{-4}	8.00×10^{-4}	6.32×10^{-7}	1	4	4	-7.46×10^{-3}	2.70×10^{-5}	-2.01×10^{-7}
3	4	4	7.90×10^{-4}	7.78×10^{-4}	6.15×10^{-7}	4	1	4	-7.46×10^{-3}	2.51×10^{-5}	-1.87×10^{-7}
4	3	3	4.47×10^{-5}	9.25×10^{-3}	4.13×10^{-7}	2	2	2	-1.79×10^{-7}	7.98×10^{-1}	-1.43×10^{-7}
3	4	3	4.47×10^{-5}	9.00×10^{-3}	4.02×10^{-7}	4	3	1	-4.22×10^{-4}	3.24×10^{-4}	-1.37×10^{-7}
3	3	4	4.47×10^{-5}	8.98×10^{-3}	4.01×10^{-7}	1	3	4	-4.22×10^{-4}	3.15×10^{-4}	-1.33×10^{-7}
3	3	3	2.52×10^{-6}	1.04×10^{-1}	2.62×10^{-7}	3	4	1	-4.22×10^{-4}	3.12×10^{-4}	-1.32×10^{-7}

-1, 100 mM NaCl, 6 mM $MgCl_2$											
Positive terms						Negative terms					
i	j	k	$\delta\bar{v}_i\delta\bar{v}_j\delta\bar{v}_k$	w_{ijk}	$w_{ijk}\delta\bar{v}_i\delta\bar{v}_j\delta\bar{v}_k$	i	j	k	$\delta\bar{v}_i\delta\bar{v}_j\delta\bar{v}_k$	w_{ijk}	$w_{ijk}\delta\bar{v}_i\delta\bar{v}_j\delta\bar{v}_k$
4	4	4	6.39×10^{-3}	2.17×10^{-4}	1.38×10^{-6}	2	2	2	-2.52×10^{-6}	1.41×10^{-1}	-3.56×10^{-7}
3	3	3	4.67×10^{-7}	6.50×10^{-1}	3.04×10^{-7}	3	3	1	-6.89×10^{-6}	1.80×10^{-2}	-1.24×10^{-7}
3	1	2	1.21×10^{-5}	4.66×10^{-3}	5.64×10^{-8}	1	3	3	-6.89×10^{-6}	1.76×10^{-2}	-1.21×10^{-7}
2	1	3	1.21×10^{-5}	4.67×10^{-3}	5.16×10^{-8}	1	2	2	-2.12×10^{-5}	5.02×10^{-3}	-1.06×10^{-7}
3	1	1	1.02×10^{-4}	5.04×10^{-4}	5.13×10^{-8}	2	2	1	-2.12×10^{-5}	4.69×10^{-3}	-9.94×10^{-8}
1	1	3	1.02×10^{-4}	4.93×10^{-4}	5.02×10^{-8}	3	1	3	-6.89×10^{-6}	1.42×10^{-2}	-9.80×10^{-8}
1	3	1	1.02×10^{-4}	4.86×10^{-4}	4.95×10^{-8}	1	2	1	-1.78×10^{-4}	1.67×10^{-4}	-2.97×10^{-8}
2	2	3	1.44×10^{-6}	2.93×10^{-2}	4.21×10^{-8}	2	1	2	-2.12×10^{-5}	1.40×10^{-3}	-2.97×10^{-8}

-2, 300 mM NaCl, 6 mM $MgCl_2$											
Positive terms						Negative terms					
i	j	k	$\delta\bar{v}_i\delta\bar{v}_j\delta\bar{v}_k$	w_{ijk}	$w_{ijk}\delta\bar{v}_i\delta\bar{v}_j\delta\bar{v}_k$	i	j	k	$\delta\bar{v}_i\delta\bar{v}_j\delta\bar{v}_k$	w_{ijk}	$w_{ijk}\delta\bar{v}_i\delta\bar{v}_j\delta\bar{v}_k$
3	3	3	7.26×10^{-5}	2.79×10^{-2}	2.02×10^{-6}	1	1	1	-4.11×10^{-4}	9.08×10^{-4}	-3.73×10^{-7}
4	4	4	6.37×10^{-3}	3.06×10^{-4}	1.95×10^{-6}	1	2	2	-2.02×10^{-6}	7.75×10^{-2}	-1.56×10^{-7}
1	2	1	2.88×10^{-5}	8.39×10^{-3}	2.42×10^{-7}	2	2	1	-2.02×10^{-6}	7.75×10^{-2}	-1.56×10^{-7}
1	1	2	2.88×10^{-5}	8.38×10^{-3}	2.42×10^{-7}	2	1	2	-2.02×10^{-6}	7.74×10^{-2}	-1.56×10^{-7}
2	1	1	2.88×10^{-5}	8.38×10^{-3}	2.42×10^{-7}	4	4	1	-2.56×10^{-3}	5.46×10^{-5}	-1.40×10^{-7}
2	2	2	1.42×10^{-7}	7.16×10^{-1}	1.01×10^{-7}	1	4	4	-2.56×10^{-3}	5.46×10^{-5}	-1.40×10^{-7}
4	4	2	1.79×10^{-4}	4.06×10^{-4}	7.28×10^{-8}	4	1	2	-7.18×10^{-5}	1.23×10^{-4}	-8.86×10^{-9}
2	4	4	1.79×10^{-4}	4.06×10^{-4}	7.28×10^{-8}	2	1	4	-7.18×10^{-5}	1.23×10^{-4}	-8.86×10^{-9}

-2, 100 mM NaCl, 6 mM $MgCl_2$											
Positive terms						Negative terms					
i	j	k	$\delta\bar{v}_i\delta\bar{v}_j\delta\bar{v}_k$	w_{ijk}	$w_{ijk}\delta\bar{v}_i\delta\bar{v}_j\delta\bar{v}_k$	i	j	k	$\delta\bar{v}_i\delta\bar{v}_j\delta\bar{v}_k$	w_{ijk}	$w_{ijk}\delta\bar{v}_i\delta\bar{v}_j\delta\bar{v}_k$
4	4	4	5.26×10^{-3}	4.21×10^{-4}	2.21×10^{-6}	2	2	2	-2.80×10^{-5}	8.77×10^{-2}	-2.46×10^{-6}
2	2	4	1.60×10^{-4}	1.37×10^{-2}	2.20×10^{-6}	2	4	4	-9.18×10^{-4}	2.39×10^{-3}	-2.19×10^{-6}
4	2	2	1.60×10^{-4}	1.37×10^{-2}	2.20×10^{-6}	4	4	2	-9.18×10^{-4}	2.39×10^{-3}	-2.19×10^{-6}
2	4	2	1.60×10^{-4}	1.36×10^{-2}	2.18×10^{-6}	4	2	4	-9.18×10^{-4}	2.14×10^{-3}	-1.97×10^{-6}
4	1	2	4.04×10^{-4}	3.97×10^{-3}	1.61×10^{-6}	1	2	2	-7.06×10^{-5}	2.30×10^{-2}	-1.63×10^{-6}
2	1	4	4.04×10^{-4}	3.97×10^{-3}	1.61×10^{-6}	2	2	1	-7.06×10^{-5}	2.30×10^{-2}	-1.63×10^{-6}
2	4	1	4.04×10^{-4}	3.97×10^{-3}	1.60×10^{-6}	4	4	1	-2.32×10^{-3}	6.98×10^{-4}	-1.62×10^{-6}
1	4	2	4.04×10^{-4}	3.97×10^{-3}	1.60×10^{-6}	1	4	4	-2.32×10^{-3}	6.98×10^{-4}	-1.62×10^{-6}

-2, 20 mM NaCl, 6 mM $MgCl_2$											
Positive terms						Negative terms					
i	j	k	$\delta\bar{v}_i\delta\bar{v}_j\delta\bar{v}_k$	w_{ijk}	$w_{ijk}\delta\bar{v}_i\delta\bar{v}_j\delta\bar{v}_k$	i	j	k	$\delta\bar{v}_i\delta\bar{v}_j\delta\bar{v}_k$	w_{ijk}	$w_{ijk}\delta\bar{v}_i\delta\bar{v}_j\delta\bar{v}_k$
4	4	4	6.13×10^{-3}	9.54×10^{-4}	5.85×10^{-6}	4	4	1	-3.90×10^{-3}	6.69×10^{-5}	-2.61×10^{-7}
3	3	3	1.34×10^{-4}	4.17×10^{-2}	5.59×10^{-6}	1	4	4	-3.90×10^{-3}	6.69×10^{-5}	-2.61×10^{-7}
4	4	2	5.58×10^{-5}	7.83×10^{-4}	4.37×10^{-8}	1	1	1	-1.58×10^{-3}	6.56×10^{-5}	-1.04×10^{-7}
2	4	4	5.58×10^{-5}	7.82×10^{-4}	4.36×10^{-8}	3	3	1	-3.05×10^{-4}	8.68×10^{-5}	-2.65×10^{-8}
1	2	1	2.26×10^{-5}	1.53×10^{-3}	3.46×10^{-8}	1	3	3	-3.05×10^{-4}	4.11×10^{-5}	-1.25×10^{-8}
1	1	2	2.26×10^{-5}	1.53×10^{-3}	3.46×10^{-8}	1	2	2	-3.23×10^{-7}	3.58×10^{-2}	-1.16×10^{-8}
2	1	1	2.26×10^{-5}	1.53×10^{-3}	3.45×10^{-8}	2	2	1	-3.23×10^{-7}	3.58×10^{-2}	-1.16×10^{-8}
4	1	1	2.48×10^{-3}	5.17×10^{-6}	1.28×10^{-8}	2	1	2	-3.23×10^{-7}	3.58×10^{-2}	-1.15×10^{-8}

-2, 20 mM NaCl, 0 mM $MgCl_2$											
Positive terms						Negative terms					
i	j	k	$\delta\bar{v}_i\delta\bar{v}_j\delta\bar{v}_k$	w_{ijk}	$w_{ijk}\delta\bar{v}_i\delta\bar{v}_j\delta\bar{v}_k$	i	j	k	$\delta\bar{v}_i\delta\bar{v}_j\delta\bar{v}_k$	w_{ijk}	$w_{ijk}\delta\bar{v}_i\delta\bar{v}_j\delta\bar{v}_k$
4	4	4	1.39×10^{-3}	1.34×10^{-2}	1.86×10^{-5}	4	4	1	-9.88×10^{-4}	1.34×10^{-2}	-1.32×10^{-5}
1	4	1	7.03×10^{-4}	1.34×10^{-2}	9.42×10^{-6}	1	4	4	-9.88×10^{-4}	1.34×10^{-2}	-1.32×10^{-5}
4	1	1	7.03×10^{-4}	1.34×10^{-2}	9.42×10^{-6}	4	1	4	-9.88×10^{-4}	1.34×10^{-2}	-1.32×10^{-5}
1	1	4	7.03×10^{-4}	1.34×10^{-2}	9.42×10^{-6}	1	1	1	-5.00×10^{-4}	1.34×10^{-2}	-6.70×10^{-6}
5	5	5	5.96×10^{-3}	5.56×10^{-4}	3.31×10^{-6}	2	4	4	-2.51×10^{-4}	1.03×10^{-2}	-2.58×10^{-6}
3	4	4	6.57×10^{-4}	3.59×10^{-3}	2.36×10^{-6}	4	4	2	-2.51×10^{-4}	1.03×10^{-2}	-2.58×10^{-6}
4	4	3	6.57×10^{-4}	3.59×10^{-3}	2.36×10^{-6}	2	2	2	-8.15×10^{-6}	3.14×10^{-1}	-2.56×10^{-6}
2	1	4	1.78×10^{-4}	1.03×10^{-2}	1.84×10^{-6}	3	1	4	-4.67×10^{-4}	3.59×10^{-3}	-1.68×10^{-6}

Table S5. Optimized forward and backward inverse rate constants corresponding to the elementary reactive steps for the samples studied.

Sample	k_{12}^{-1}	k_{21}^{-1}	k_{13}^{-1}	k_{31}^{-1}	k_{14}^{-1}	k_{41}^{-1}	k_{23}^{-1}	k_{32}^{-1}	k_{24}^{-1}	k_{42}^{-1}	k_{34}^{-1}	k_{43}^{-1}	k_{15}^{-1}	k_{51}^{-1}	k_{25}^{-1}	k_{52}^{-1}	k_{35}^{-1}	k_{53}^{-1}	k_{45}^{-1}	k_{54}^{-1}
+1, 300 mM NaCl	66.3	533	-	-	-	-	-	-	-	-	11.8e4	363	0.85	1.75	-	-	556	9.91	-	-
+1, 100 mM NaCl	-	-	-	-	4.97	4.86	66.1e3	548	17.7e2	95.4	1.84	0.28	-	-	-	-	-	-	-	-
+1, 20 mM NaCl	0.55	1.64	-	-	16.4e2	15.4	27.1e3	627	-	-	-	-	-	-	-	-	-	-	-	-
+1, 20 mM NaCl, 0 mM $MgCl_2$	-	-	4.64	121	-	-	25.1e2	109	731	368	4.31	0.37	-	-	-	-	-	-	-	-
-1, 100 mM NaCl	4.27	39.0	1.45	41.2	77.6e2	82.7	433	11.2e4	-	-	-	-	-	-	-	-	-	-	-	-
-2, 300 mM NaCl	0.40	3.74	-	-	539	10.8	21.8e3	518	-	-	-	-	-	-	-	-	-	-	-	-
-2, 100 mM NaCl	2.20	8.32	-	-	0.71	0.42	109	382	35.0e3	78.6e3	-	-	-	-	-	-	-	-	-	-
-2, 20 mM NaCl	0.55	12.9	52.5e3	494	180	15.6	84.5e2	18.6e2	-	-	-	-	-	-	-	-	-	-	-	-
-2, 20 mM NaCl, 0 mM $MgCl_2$	-	-	10.4	12.7	0.0005	0.0005	4.65	1.17	-	-	75.0e3	91.7e3	87.2e3	285	-	-	-	-	-	-

Inverse rate constants defined in Eq. (4), listed in units of milliseconds. Error bars are < 1% of reported values (see Statistical Uncertainty Analysis below).

Table S6. Relative free energy minima.

Sample	G_1	G_2	G_3	G_4	G_5
+1, 300 mM NaCl	3.8	2.9	0	5.7	3.8
+1, 100 mM NaCl	3.7	0	1.8	3.7	-
+1, 20 mM NaCl	1.1	0	3.6	5.9	-
+1, 20 mM NaCl, 0 mM $MgCl_2$	4.4	0	1.6	4.4	-
-1, 100 mM NaCl	3.6	1.2	0	5.5	-
-2, 300 mM NaCl	2.2	0	3.8	6.8	-
-2, 100 mM NaCl	2.5	1.3	0	3.2	-
-2, 20 mM NaCl	3.1	0	3.1	5.7	-
-2, 20 mM NaCl, 0 mM $MgCl_2$	1.6	0	1.4	1.6	6.4

The ground state is taken to be the macrostate with minimum free energy, G_{min}, and excited state energies are given by $G_i = -k_B T \ln\left(p_i^{eq}/\sigma_i\sqrt{2\pi}\right) - G_{min}$ with parameters σ_i and p_i^{eq} listed in Table S1 and Table S2, respectively. Energies are listed in units of $k_B T$.

Table S7. Free energies of activation corresponding to the elementary reactive steps.

Sample	$G^{\ddagger}_{12}$	$G^{\ddagger}_{21}$	$G^{\ddagger}_{13}$	$G^{\ddagger}_{31}$	$G^{\ddagger}_{14}$	$G^{\ddagger}_{41}$	$G^{\ddagger}_{23}$	$G^{\ddagger}_{32}$	$G^{\ddagger}_{24}$	$G^{\ddagger}_{42}$	$G^{\ddagger}_{34}$	$G^{\ddagger}_{43}$	$G^{\ddagger}_{15}$	$G^{\ddagger}_{51}$	$G^{\ddagger}_{25}$	$G^{\ddagger}_{52}$	$G^{\ddagger}_{35}$	$G^{\ddagger}_{53}$	$G^{\ddagger}_{45}$	$G^{\ddagger}_{54}$
+1, 300 mM NaCl	4.4	6.4	-	-	-	-	-	-	-	-	11.8	6.1	0	0.7	-	-	6.5	6.2	-	-
+1, 100 mM NaCl	-	-	-	-	2.9	2.9	12.4	7.6	8.8	1.9	1.9	0	-	-	-	-	-	-	-	-
+1, 20 mM NaCl	0	1.1	-	-	8.0	3.2	10.8	7.0	-	-	-	-	-	-	-	-	-	-	-	-
+1, 20 mM NaCl, 0 mM $MgCl_2$	-	-	2.5	5.8	-	-	8.8	5.7	7.6	6.9	2.4	0	-	-	-	-	-	-	-	-
-1, 100 mM NaCl	1.1	3.3	0	3.3	8.6	4.0	5.7	11.2	-	-	-	-	-	-	-	-	-	-	-	-
-2, 300 mM NaCl	0	2.2	-	-	7.2	3.3	10.9	7.1	-	-	-	-	-	-	-	-	-	-	-	-
-2, 100 mM NaCl	1.7	3.0	-	-	0.5	0	5.5	6.8	-	-	-	-	-	-	-	-	-	-	-	-
-2, 20 mM NaCl	0	3.2	11.5	6.8	5.8	3.3	9.6	8.1	-	-	-	-	-	-	-	-	-	-	-	-
-2, 20 mM NaCl, 0 mM $MgCl_2$	-	-	9.9	10.1	0	0	9.1	7.8	-	-	18.8	19.0	19.0	13.2	-	-	-	-	-	-

The activation energies are determined from $G^{\ddagger}_{ij} = -k_B T \ln(k_{ij}/k_{max})$ where k_{max} is the fastest rate constant determined from the analysis. Energies are listed in units of $k_B T$.

Table S8. Free energies of activation corresponding to the elementary reactive steps.

Sample	$G^{o\ddagger}_{12}$	$G^{o\ddagger}_{21}$	$G^{o\ddagger}_{13}$	$G^{o\ddagger}_{31}$	$G^{o\ddagger}_{14}$	$G^{o\ddagger}_{41}$	$G^{o\ddagger}_{23}$	$G^{o\ddagger}_{32}$	$G^{o\ddagger}_{24}$	$G^{o\ddagger}_{42}$	$G^{o\ddagger}_{34}$	$G^{o\ddagger}_{43}$	$G^{o\ddagger}_{15}$	$G^{o\ddagger}_{51}$	$G^{o\ddagger}_{25}$	$G^{o\ddagger}_{52}$	$G^{o\ddagger}_{35}$	$G^{o\ddagger}_{53}$	$G^{o\ddagger}_{45}$	$G^{o\ddagger}_{54}$
+1, 300 mM NaCl	8.2	9.3	-	-	-	-	-	-	-	-	11.8	11.8	3.8	4.5	-	-	6.5	6.2	-	-
+1, 100 mM NaCl	-	-	-	-	6.6	6.6	12.4	9.4	8.8	9.5	3.7	3.7	-	-	-	-	-	-	-	-
+1, 20 mM NaCl	1.1	1.1	-	-	9.1	9.1	10.8	10.6	-	-	-	-	-	-	-	-	-	-	-	-
+1, 20 mM NaCl, 0 mM $MgCl_2$	-	-	6.9	7.4	-	-	8.8	7.3	7.6	11.3	4.0	4.4	-	-	-	-	-	-	-	-
-1, 100 mM NaCl	4.7	4.5	3.6	3.3	12.2	9.5	6.9	11.2	-	-	-	-	-	-	-	-	-	-	-	-
-2, 300 mM NaCl	2.2	2.2	-	-	9.4	10.1	10.9	10.9	-	-	-	-	-	-	-	-	-	-	-	-
-2, 100 mM NaCl	4.2	4.3	-	-	3.0	3.2	6.8	6.8	-	-	-	-	-	-	-	-	-	-	-	-
-2, 20 mM NaCl	3.1	3.2	14.6	9.9	8.9	9.0	9.6	11.2	-	-	-	-	-	-	-	-	-	-	-	-
-2, 20 mM NaCl, 0 mM $MgCl_2$	-	-	11.5	11.5	1.6	1.6	9.1	9.2	-	-	20.2	20.6	20.6	19.6	-	-	-	-	-	-

The activation energies are determined from $G^{0\ddagger}_{ij} = -k_B T \ln(k_{ij}/k_{max}) + G_i$ where k_{max} is the fastest rate constant determined from the analysis and G_i is the free energy minimum of the *i*th macrostate. Energies are listed in units of $k_B T$.

Salt concentration dependence of the local conformational macrostates of the +1 and -2 $(iCy3)_2$ dimer-labeled ss-dsDNA fork constructs. In Fig. S1 we present our salt-concentration-dependent results for the +1 $(iCy3)_2$ dimer-labeled ss-dsDNA construct. At the highest salt concentrations that we studied (300 mM NaCl, 6 mM $MgCl_2$; Fig. S1*A*), nearly all the population resides in macrostate S_3 (LHU, p_3^{eq} = 0.91), and the remaining populations in macrostates S_1 (PO), S_2 (WC), S_4 (RHU) and S_5 (to be assigned). The two-point TCF exhibits a somewhat faster overall decay [with mean relaxation time $\bar{\tau}$ $(= \sum_i \alpha_i t_i)$ = 25 ms] compared to the 'physiological' salt sample ($\bar{\tau}$ = 35 ms, Fig. S1*B*). However, the three-point TCF exhibits significantly greater initial amplitude at the resolution of T_w = 10 ms, indicating the presence of highly correlated three-point transition pathways at elevated salt concentrations. Indeed, Table S4 of the SI shows that the largest 'pathway terms' at T_w = 10 ms involve recurrent measurements of macrostates S_5 and S_4 (i.e., $\delta\bar{v}_5\delta\bar{v}_5\delta\bar{v}_5$ and $\delta\bar{v}_4\delta\bar{v}_4\delta\bar{v}_4$) and transitions between macrostates S_1 and S_5 (e.g., $\delta\bar{v}_1\delta\bar{v}_1\delta\bar{v}_5$, $\delta\bar{v}_5\delta\bar{v}_1\delta\bar{v}_5$, etc.). The CPs (bottom row) show that macrostate S_5 is a transient intermediate species; the transition probability p_{15} increases in the sub-millisecond time scales, reaches its maximum value at ~20 ms, and decays to its equilibrium value, p_5^{eq} = 0.02, with a time scale of ~100 ms. The optimized kinetic network scheme shown in Fig. S2*A* indicates that macrostate S_5 is directly connected to macrostates S_1 and S_3, and the free energy minimum of S_5 has an equal value to that of S_1 (see Table S6 of the SI).

At 'physiological' salt concentrations (100 mM NaCl, 6 mM $MgCl_2$, Fig. S1*B*), the major component is macrostate S_2 (WC, p_2^{eq} = 0.82) and the second most populated component is macrostate S_3 (LHU, p_3^{eq} = 0.14). When the salt concentration is decreased below 'physiological' (20 mM NaCl and 6 mM $MgCl_2$, Fig. S1*C*), the major component is macrostate S_2 (WC, p_2^{eq} = 0.74), and the second most populated component is S_1 (PO, p_1^{eq} = 0.24). At this reduced salt concentration, the S_3 macrostate exists as a trace population (p_3^{eq} = 0.02). The two-point TCF exhibits faster overall dynamics at these low salt concentrations ($\bar{\tau}$ = 13 ms), which is primarily due to significant lowering of the transition barriers between the two most thermodynamically favored macrostates, S_2 (WC) and S_1 (PO) (Fig. S2*E* and S2*F*). Table S4 shows that the largest pathway terms contributing to the initial amplitude of the three-point TCF involve recurrent measurements of macrostates S_1, S_3 and S_4 and transitions between macrostates S_1 and S_2.

Finally, elimination of divalent magnesium ions at low monovalent sodium ion concentration (20 mM NaCl, 0 mM $MgCl_2$, Fig. S1*D*) leads to a reversal in the relative stabilities of macrostates S_1 and S_3 (see Figs. S2*G* and S2*H*), resulting in a pattern that significantly resembles that seen under 'physiological' salt conditions. This comparison, with and without magnesium ions, permits us to isolate the effects of magnesium near the +1 position. When magnesium is eliminated at low sodium concentration, the major component is macrostate S_2 (WC, p_2^{eq} = 0.82) and the second most populated component is macrostate S_3 (LHU, p_3^{eq} = 0.16). In the absence of magnesium, macrostate S_1 (PO) exists as a trace population (p_1^{eq} = 0.01). The two-point TCF exhibits faster overall dynamics in comparison to the physiological sample ($\bar{\tau}$ = 17 ms), although somewhat slower than in the presence of magnesium. The very small initial amplitude of the three-point TCF (Fig. S1*D*) indicates a near cancellation between positive and negative three-point pathway terms (see Table S3). Finally, the CPs at physiological and zero magnesium concentrations (Figs. S1*B* and S1*D*, respectively) show that macrostate S_3 (LHU) is a transient intermediate under these conditions.

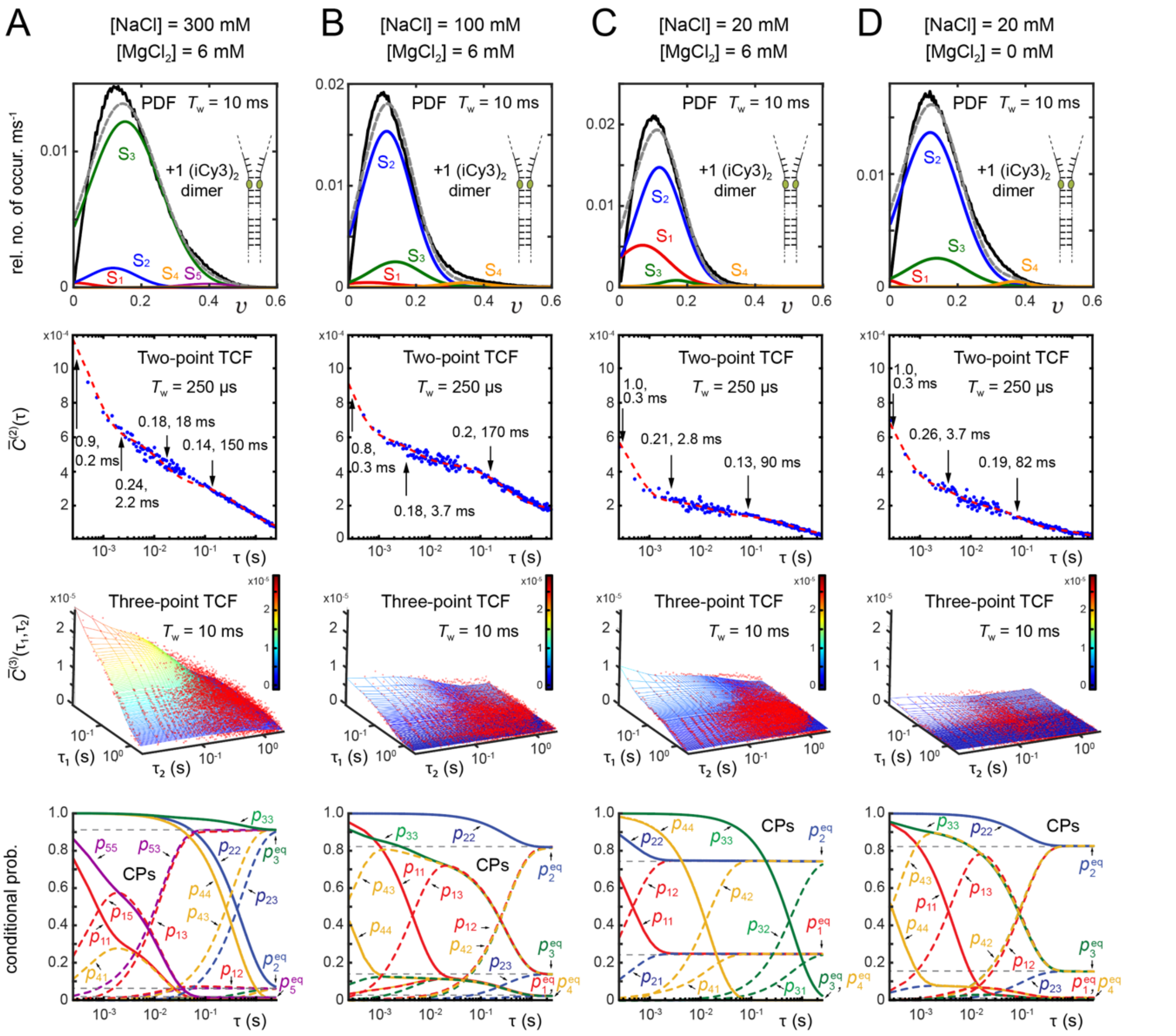

Figure S1. Results of kinetic network model analyses applied to PS-SMF measurements of the +1 $(iCy3)_2$ dimer-labeled ss-dsDNA construct in 10 mM Tris at pH 8.0 and (***A***) 300 mM NaCl, 6 mM $MgCl_2$; (***B***) 100 mM NaCl, 6 mM $MgCl_2$; (***C***) 20 mM NaCl, 6 mM $MgCl_2$; (***D***) 20 mM NaCl, 0 mM $MgCl_2$. Formatting and color schemes are the same as described in Fig. 6 of the main text. Optimized values of the Gaussian parameters are listed in Table S1 and Table S2.

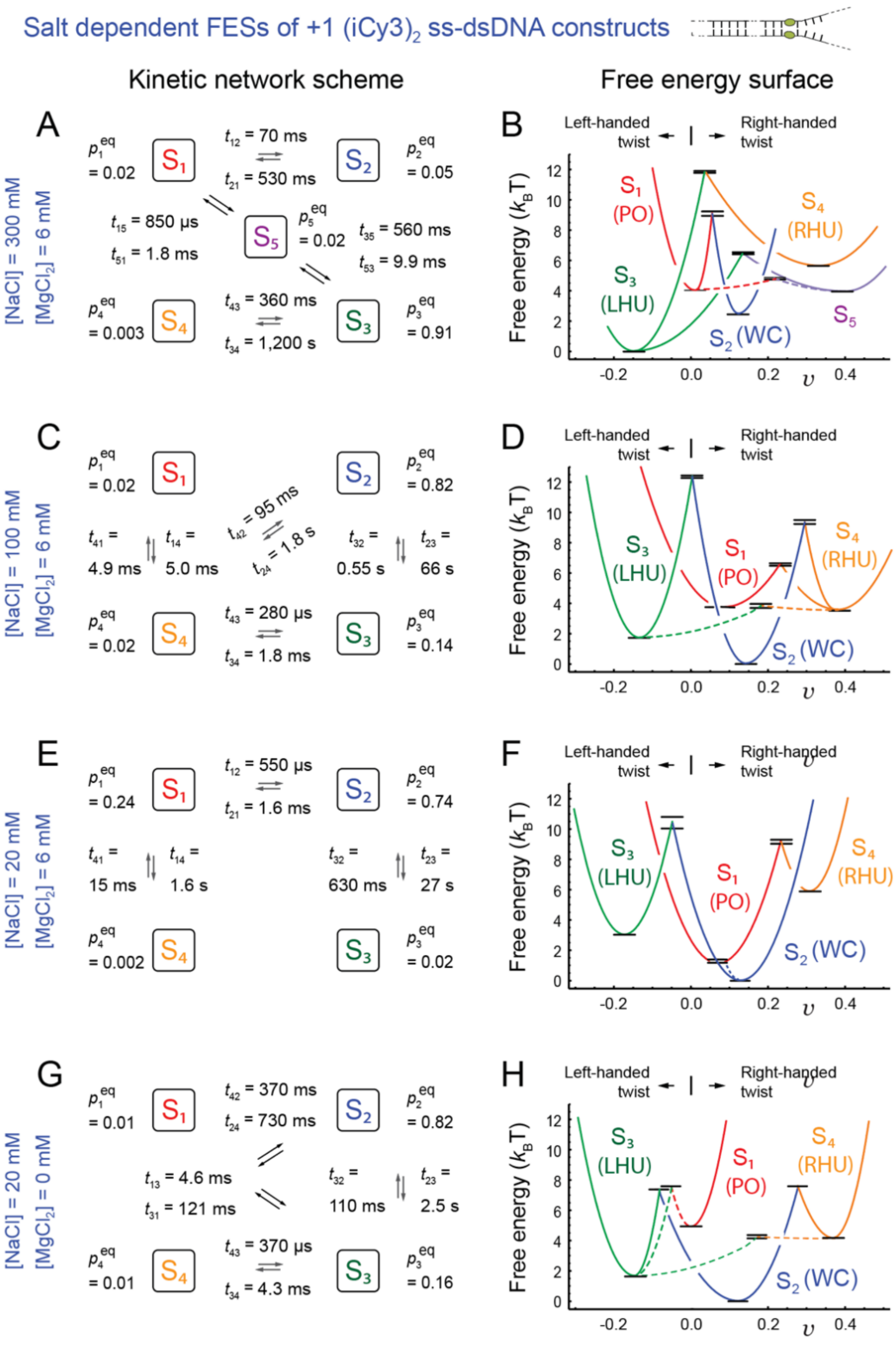

Figure S2. Kinetic network schemes (***A***, ***C***, ***E***, ***G***) and free energy surfaces (FESs) (***B***, ***D***, ***F***, ***H***) from the analyses of the +1 (iCy3)$_2$ dimer-labeled ss-dsDNA fork construct in 10 mM Tris at pH 8.0 and (***A***, ***B***) 300 mM NaCl, 6 mM $MgCl_2$; (***C***, ***D***) 100 mM NaCl, 6 mM $MgCl_2$; (***E***, ***F***) 20 mM NaCl, 6 mM $MgCl_2$; (***G***, ***H***) 20 mM NaCl, 0 mM $MgCl_2$. Formatting and color schemes are the same as described in Fig. 7 of the main text. Optimized values of the time constants are listed in Table S5, free energy minima in Table S6, and transition energies in Table S7.

In Fig. S3, we present our salt concentration-dependent results of the -2 $(iCy3)_2$ dimer-labeled ss-dsDNA fork construct. The optimized kinetic network schemes for each set of salt concentrations and the associated FESs are presented in Fig. S4. As discussed in Sect. 3.1, at the 'physiological' salt concentrations (100 mM NaCl, 6 mM $MgCl_2$, Fig. S3*B*) the major component is macrostate S_3 (LHU, p_3^{eq} = 0.71) and the second most populated component is macrostate S_2 (WC, p_2^{eq} = 0.20), which is opposite to the population order observed for the +1 construct. The two-point TCF exhibits a relatively fast overall decay ($\bar{\tau}$ = 12 ms), indicating that the transition barriers are relatively low in comparison to the +1 construct. Moreover, the three-point TCF exhibits a weak and negative initial amplitude at resolution T_w = 10 ms, which is consistent with relatively low transition barriers between macrostates. The effects of increasing or decreasing salt concentrations on the -2 construct are opposite to those we observed with the +1 construct. At the highest salt concentrations (300 mM NaCl, 6 mM $MgCl_2$; Fig. S3*A*), the major component is macrostate S_2 (WC, p_2^{eq} = 0.88) and the second component is macrostate S_1 (PO, p_1^{eq} = 0.10). Macrostates S_3 (LHU) and S_4 (RHU) are populated at trace levels at these high salt concentrations. The two-point TCF exhibits a slower overall decay ($\bar{\tau}$ = 25 ms) compared to 'physiological' concentrations, indicating that at least some transition barriers are elevated.

Decreasing the monovalent sodium ion concentration from the 'physiological' value to 20 mM NaCl and 6 mM $MgCl_2$, (Fig. S3*C*), resulted in the transfer of nearly all the population to macrostate S_2 (p_2^{eq} = 0.92), with only trace populations occurring in macrostates S_1, S_3 and S_4. The two-point TCF at the lower salt concentrations decays relatively rapidly ($\bar{\tau}$ = 14 ms), albeit slightly slower than under 'physiological' conditions. Elimination of divalent magnesium ions at the low sodium ion concentration significantly alters the equilibrium distribution of macrostates. At these lowest salt concentrations (20 mM NaCl, 0 mM $MgCl_2$, Fig. S3*D*), the major component is macrostate S_2 (WC, p_2^{eq} = 0.62) and the remaining population is shared nearly equally between macrostates S_1 (PO, p_1^{eq} = 0.12), S_3 (LHU, p_3^{eq} = 0.15) and S_4 (RHU, p_4^{eq} = 0.12). A trace population is also present in macrostate S_5. The two-point TCF at the lowest salt concentrations exhibits a significantly slower overall decay ($\bar{\tau}$ = 85 ms) than for any of the other constructs and salt conditions that we studied. The slow decay of the two-point TCF indicates that the transition barriers are much higher than for the other samples, which is corroborated by the relatively large and positive initial amplitude of the three-point TCF at resolution T_w = 10 ms. Table S2 of the SI

shows that the three-point pathway terms that dominate on the shortest time scales involve transitions between macrostates S_1 and S_4, and the optimized network scheme shown in Fig. S4*G* indicates that nearly all five of the macrostates may directly interconvert between one another.

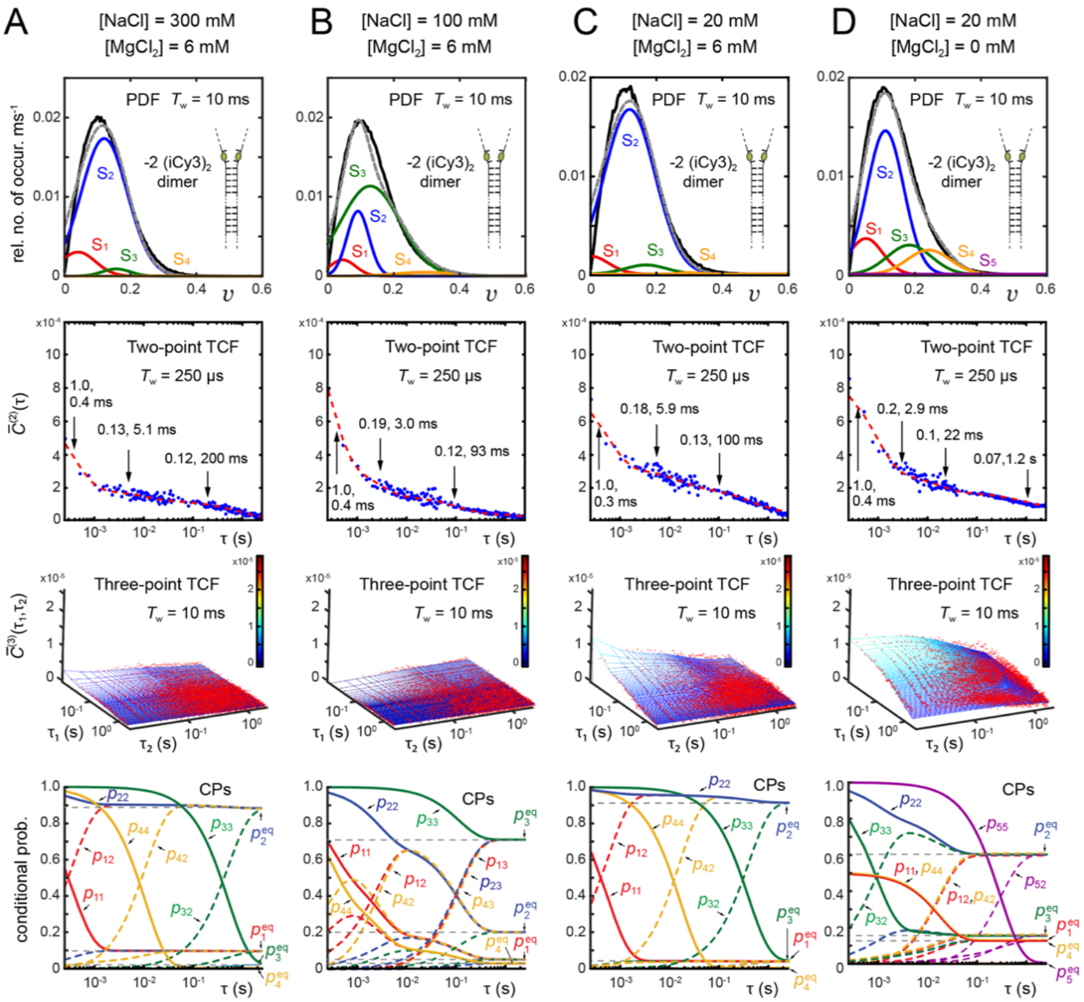

Figure S3. Results of kinetic network model analyses applied to PS-SMF measurements of the -2 $(iCy3)_2$ dimer-labeled ss-dsDNA construct in 10 mM Tris at pH 8.0 and (***A***) 300 mM NaCl, 6 mM $MgCl_2$; (***B***) 100 mM NaCl, 6 mM $MgCl_2$; (***C***) 20 mM NaCl, 6 mM $MgCl_2$; (***D***) 20 mM NaCl, 0 mM $MgCl_2$. Formatting and color schemes are the same as those in Fig. 6 of the main text.

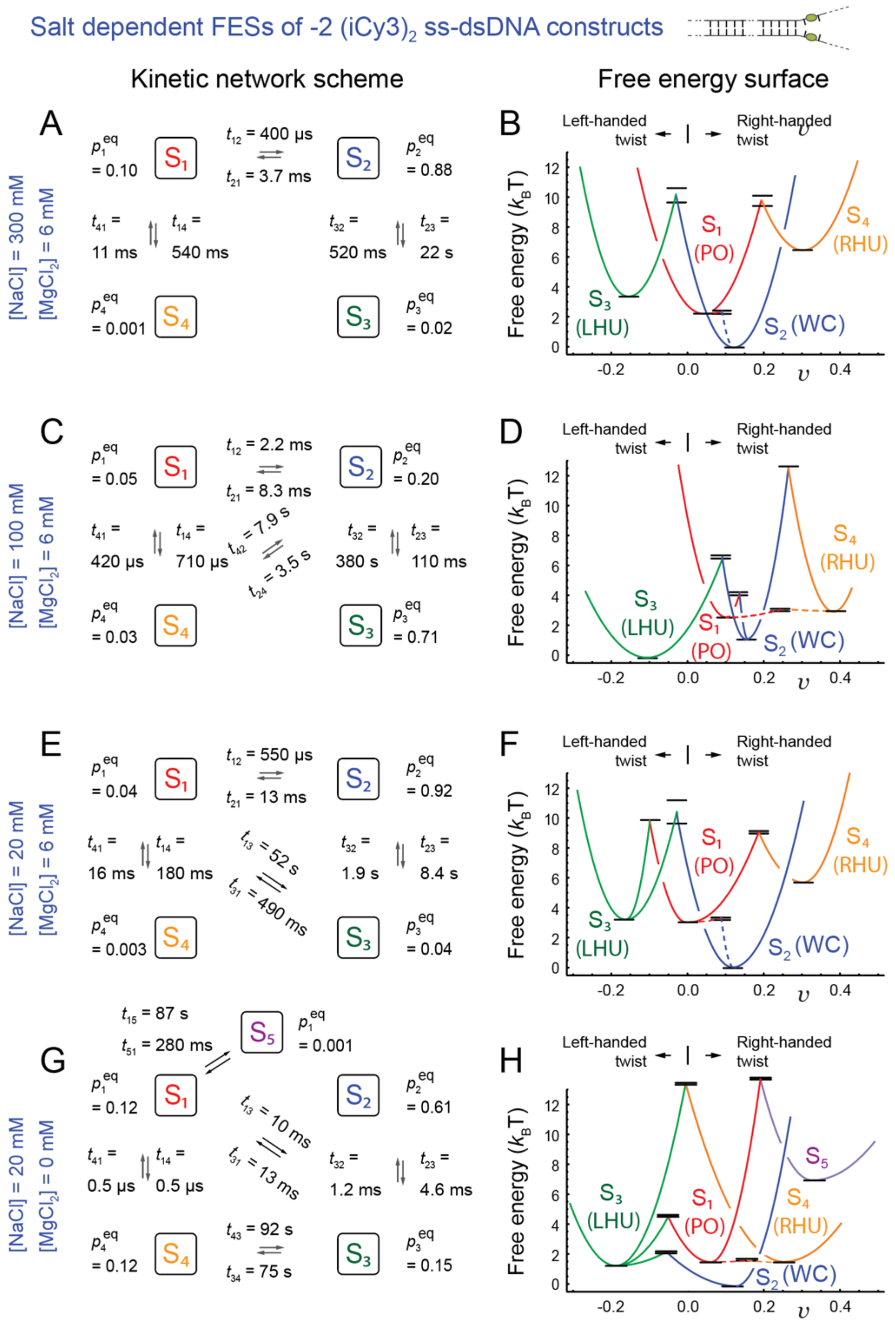

Figure S4. Kinetic network schemes (***A***, ***C***, ***E***, ***G***) and free energy surfaces (FESs) (***B***, ***D***, ***F***, ***H***) from the analyses of the -2 $(iCy3)_2$ dimer-labeled ss-dsDNA fork construct in 10 mM Tris at pH 8.0 and (***A***, ***B***) 300 mM NaCl, 6 mM $MgCl_2$; (***C***, ***D***) 100 mM NaCl, 6 mM $MgCl_2$; (***E***, ***F***) 20 mM NaCl, 6 mM $MgCl_2$; (***G***, ***H***) 20 mM NaCl, 0 mM $MgCl_2$. Formatting and color schemes are the same as those in Fig. 7 of the main text.

Iterative multiparameter optimization procedure. We performed an iterative multi-parameter optimization procedure, like the one described by Phelps *et al.* (1,2) and Israels *et al.* (3), to find the set of time constants and equilibrium probabilities that best match the experimentally-derived PDF [$P(\nu)$], two-point TCF [$\bar{C}^{(2)}(\tau)$, C2 function] and three-point TCF [$\bar{C}^{(3)}(\tau_1, \tau_2)$, C3 function]. To assess the quality of the agreement between the solutions to the master equation and the statistical functions constructed from our experimental data, we introduced the global least squares error function χ^2_{tot}, as described in Sect. 2.5 of the main text. For each of the three statistical functions, q [= PDF, C2, C3], we define the component error function

$$\chi^{2\,(q)} = \Sigma_i \frac{\left[\psi^{(q)}(i) - \bar{\psi}^{(q)}(i)\right]^2}{\psi^{(q)}(i)} \tag{S1}$$

where $\psi^{(q)}(i)$ and $\bar{\psi}^{(q)}(i)$ are the experimental and simulated functions, respectively, and the index i enumerates the data points within the coordinate space of the qth function.

The total error function, χ^2_{tot}, is the sum of weighted contributions from the three statistical functions.

$$\chi^2_{tot} = \alpha_{PDF}\chi^{2\,(PDF)} + \alpha_{c2}\,\chi^{2\,(C2)}_{wghtd} + \alpha_{C3}\,\chi^{2\,(C3)}_{wghtd} \tag{S2}$$

In Eq. (S2), the parameter α_q is a scaling factor that balances the relative contribution from each statistical function. Typical magnitudes (i.e., maxima) of the PDF, C2 and C3 functions are ~10^{-1}, ~10^{-3} and ~10^{-5}, respectively. The factors α_q were thus adjusted to ensure that each of the terms in Eq. (S2) contributed equally to the globally minimized value of χ^2_{tot}, which reflects optimal agreement between all three simulated and experimental functions.

In addition to the above considerations, it was necessary to account for the time-dependent variations in the data point densities, signal amplitudes and noise of the C2 and C3 functions, which each span multiple decades in their respective time variables. To optimize the agreement between simulated and experimental functions over the full range of time scales, we used Eq. (S2)

to calculate the 'weighted' functions $\chi^{2\,(C2/C3)}_{wghtd}$ in which we assigned larger weights to regions of data with relatively sparse point density, high signal amplitude and low noise.

We first describe our procedure for determining $\chi^{2\,(C2)}_{wghtd}$ for the C2 function, which is a one-dimensional function of the time delay, τ. The C2 function contains N_{data} (= ~200) data points and spans N_{dec} (= ~4) decades in time. We divided each decade into N_{seg} (= ~10) 'segments,' which we enumerated using the index j. In Eq. (S3), we define the j-dependent 'inverse weight function' $\eta^{(C2)}_j$ which sums over the N_j data points contained within the jth segment.

$$\eta^{(C2)}_j = \sum_{i=1}^{N_j} \left[\psi^{(C2)}_j(i) - \varepsilon^{(C2)}_j\, \psi^{(C2)}_j(i)\right]^2 \tag{S3}$$

Here $\psi^{(C2)}_j(i)$ is the ith data point contained within the jth segment, and $\varepsilon^{(C2)}_j = \gamma^{(C2)} j + \gamma^{(C2)}_0$ is a linear function of j with constant slope $\gamma^{(C2)}$ and offset $\gamma^{(C2)}_0$. The slope $\gamma^{(C2)}$ was chosen to account for the rate of increasing point density, decreasing amplitude, and increasing noise across segments within a given decade.

Finally, we expanded the dimension of the j-dependent function $\eta^{(C2)}_j$ (with dimension $N_{seg} \cdot N_{dec}$) to include values corresponding to each of the N_{data} data points. We thus calculated the weighted error function used in Eq. (S2) according to

$$\chi^{2\,(C2)}_{wghtd} = \sum_{i=1}^{N_{data}} \left[\psi^{(C2)}(i) - \bar{\psi}^{(C2)}(i)\right]^2 \left\{\frac{\eta^{(C2)}(i)}{max\left[\eta^{(C2)}(i)\right]}\right\}^{-1} \tag{S4}$$

where the index i spans each of the data points contained within the C2 function. In Eq. (S4), the factor $\left\{\eta^{(C2)}(i)/max\left[\eta^{(C2)}(i)\right]\right\}^{-1}$ assigns a greater weight to regions of comparatively low point density, higher signal amplitude and low noise.

To determine $\chi^{2\,(C3)}_{wghtd}$ for the C3 function we performed a calculation like that for $\chi^{2\,(C2)}_{wghtd}$. However, we generalized the procedure to account for the dependence of the C3 function on the two time variables, τ_1 and τ_2. The number of data points contained by the C3 function is N^2_{data} (= ~7,500). Each time variable spans N_{dec} (= ~2.5) decades, and each decade was divided into N_{seg} (~10) segments. The two-dimensional function was thus partitioned into N^2_{seg} (~90) segments, each of which are enumerated using the indices j and j'. The two-dimensional inverse weight function is thus defined

$$\eta^{(C3)}_{jj'} = \sum_{i,i'=1}^{N_j,N_{j'}} \left[\psi^{(C3)}_{jj'}(i,i') - \varepsilon^{(C3)}_{jj'}\psi^{(C3)}_{jj'}(i,i')\right]^2 \tag{S5}$$

In Eq. (S5), $\psi^{(C3)}_{jj'}(i,i')$ is the ii'th data point contained within the jj'th segment, and $\varepsilon^{(C3)}_{jj'} = \gamma^{(C3)}jj' + \gamma^{(C3)}_0$ is a linear joint function of j and j' with constant slope $\gamma^{(C3)}$ and constant offset $\gamma^{(C3)}_0$, like that implemented for the C2 function.

We expanded the dimension of the jj'-dependent function $\eta^{(C3)}_{jj'}$ (with dimension $N^2_{seg}N^2_{dec}$) to include values corresponding to each of the N^2_{data} data points. We thus calculated the weighted error function used in Eq. (S2) according to

$$\chi^{2\,(C3)}_{weighted} = \sum_{i,i'=1}^{N_{data},N_{data}} \left[\psi^{(C3)}(i,i') - \bar{\psi}^{(C3)}(i,i')\right]^2 \left\{\frac{\eta^{(C3)}(i,i')}{max[\eta^{(C3)}(i,i')]}\right\}^{-1} \tag{S6}$$

We determined optimal values for the input parameters $\gamma^{(C2)}$, $\gamma^{(C2)}_0$, $\gamma^{(C3)}$, $\gamma^{(C3)}_0$ and N_{seg} for the weight functions $\eta^{(C2)}_j$ and $\eta^{(C3)}_{jj'}$ given by Eq. (S3) and Eq. (S5), respectively, and for the scaling factors α_q given by Eq. (S2) as an initialization phase of our optimization calculations. The sensitivity of these calculations to the most significant regions of the data was thus optimized.

We carried out our multiparameter optimization calculation in two stages. In the first stage, we used a custom-designed genetic algorithm (GA) to broadly search the parameter space and to obtain a set of parameters that produced qualitatively good agreement between simulated and experimental functions. In the second stage of the calculation, the output solutions obtained from the GA were fed into a commercial multivariable search function (*patternsearch*, MATLAB, The MathWorks, USA), which refined the solutions by further reducing the value of χ_{tot}^2. Further details of this procedure are given below.

The GA that we implemented is very similar to the one used by Israels *et al.* (3). However, to fit the C2 and C3 functions of the current work, which span several decades in time, some modifications were introduced. The GA is an optimization procedure that is inspired by biological evolution. A schematic workflow diagram for the GA is shown in Fig. S5. The calculation proceeds through a set of successive hierarchical 'iterations' (indexed by N). Within each iteration are associated multiple 'generations' (indexed by n), and within each generation are associated a population of initial 'guesses' for the set of input parameters (represented by differently colored circles in Fig. S5*A*). The input parameters are: (*i*) the mean visibilities of the macrostates, $\bar{v}_i$; (*ii*) the Gaussian widths of the macrostates, σ_i; and (*iii*) the inverse rate constants of transitions between macrostates, $k_{ij}^{-1} = t_{ij}$ where $i, j \in \{1,2,3,4\}$.

Within each iteration, the calculation proceeds through multiple sequential generations, which are 'evolved' according to the workflow outlined in Fig. S5*B* and Fig. 5S*C*. The first generation, G_1, typically contains ~2,000 initial guesses of the parameters $\bar{v}_i, t_{ij}$, and σ_i, which are each assigned values using a random number generator (see Fig. S5*B*). Because the parameters, t_{ij}, span multiple decades, their values are assigned by sampling logarithmically the full range of time scales. Each individual guess within the first generation is ranked according to its agreement with the experimental functions, which is quantified using the value of χ_{tot}^2 [Eq. (S2)]. From this relatively large initial generation (G_1) is selected the top 10% (~200) of individuals exhibiting the most favorable agreement between simulated and experimental functions.

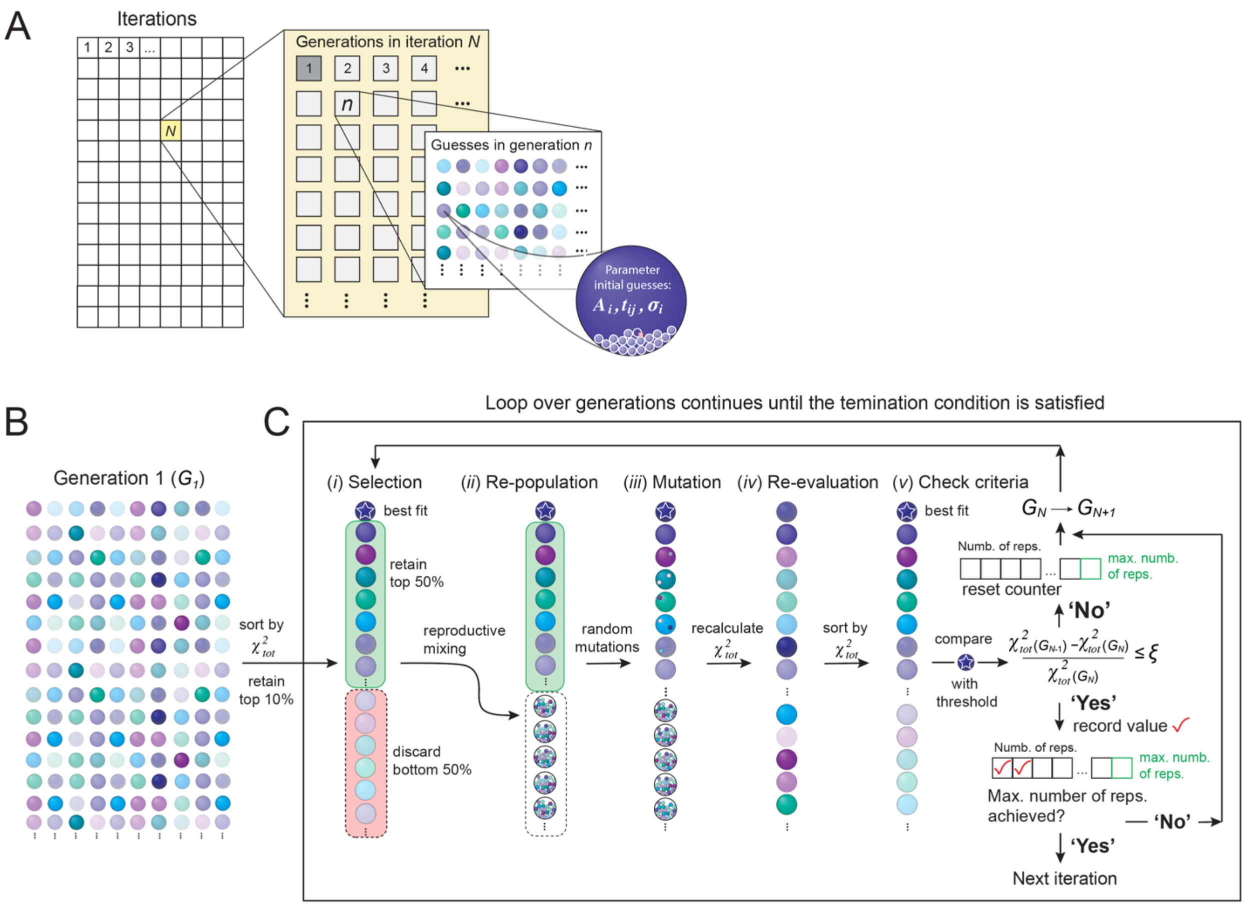

Figure S5. Workflow diagram of the multiparameter optimization procedure used in this work. See SI text for further details. Adapted from ref. (3).

The population of ~200 individuals is next refined through a series of 'evolutionary steps,' as illustrated in Fig. S5*C*. In the 'selection' step (*i*), the top 50% of individuals are retained and the bottom 50% of individuals are discarded. In the 'reproductive mixing' step (*ii*), the top ranking individuals from step (*i*) are used to create 'progeny,' which involves the random exchange (or 'mixing') of parameters between individuals. In the 'mutation' step (*iii*), the parameters of all but the top two individuals are randomly varied to generate 'diversity.' In the re-evaluation step (*iv*), the refined population is ranked by updating the values of χ^2_{tot}. In the final step (*v*), the value of χ^2_{tot} for the top individual (marked with a blue star) is compared to the highest ranked value obtained from the previous generation. When the value of the top individual has improved by an amount that meets or exceeds the set threshold value ξ (= ~0.01), the updated χ^2_{tot} value is recorded, and the refined generation is used as input to repeat the next cycle. Generational cycles

that do not satisfy the threshold criterion are tallied and their refined populations are also used as input for the next cycle, but their χ^2_{tot} values are discarded. After ~10 successive generational cycles occur for which the threshold criterion is satisfied, the final solution is recorded, and the next iteration is begun.

The GA is typically carried for a duration of 100 – 500 iterations. A subset of these solutions exhibit qualitatively good agreement between all three simulated and experimental functions. These output solutions obtained from the GA are then fed into the multivariable problem solver, *patternsearch* (MATLAB, The MathWorks, USA), to arrive at a final optimized solution.

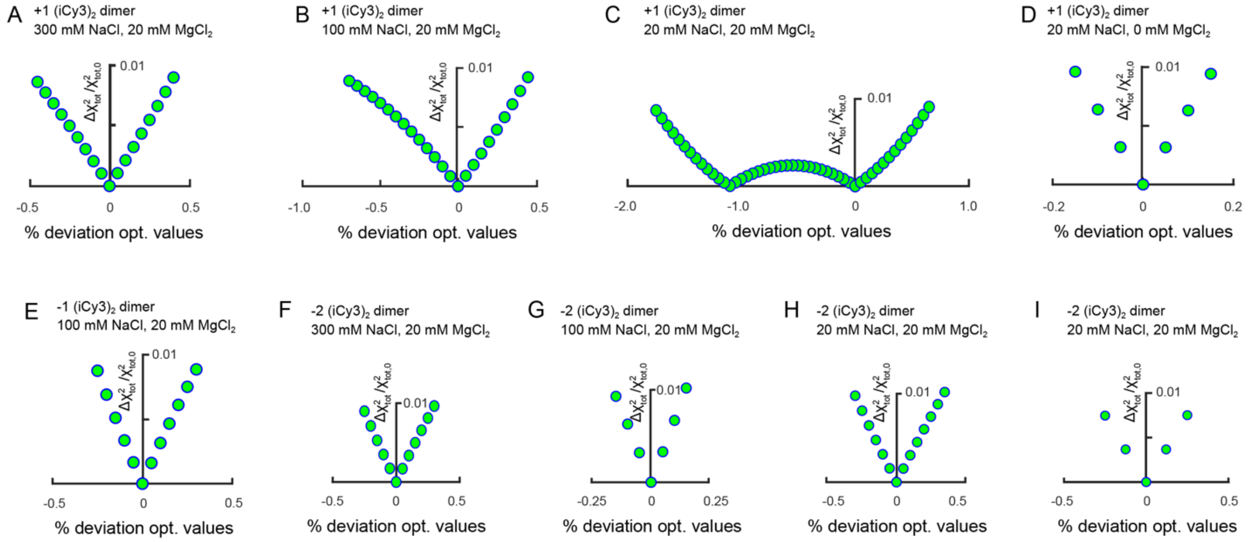

Figure S6. Relative deviation of the error function $\Delta\chi^2_{tot}/\chi^2_{tot,0}$ from the optimized value $\chi^2_{tot,0}$, as a function of the time constants and the mean visibility parameter uncertainties for each of $(iCy3)_2$ dimer-labeled ss-dsDNA constructs and salt concentrations studied in this work. (***A***) +1 $(iCy3)_2$ dimer at 300 mM NaCl and 6 mM $MgCl_2$. (***B***) +1 $(iCy3)_2$ dimer at 100 mM NaCl and 6 mM $MgCl_2$. (***C***) +1 $(iCy3)_2$ dimer at 20 mM NaCl and 6 mM $MgCl_2$. (***D***) +1 $(iCy3)_2$ dimer at 20 mM NaCl and 0 mM $MgCl_2$. (***E***) -1 $(iCy3)_2$ dimer at 100 mM NaCl and 6 mM $MgCl_2$. (***F***) -2 $(iCy3)_2$ dimer at 300 mM NaCl and 6 mM $MgCl_2$. (***G***) -2 $(iCy3)_2$ dimer at 100 mM NaCl and 6 mM $MgCl_2$. (***H***) -2 $(iCy3)_2$ dimer at 20 mM NaCl and 6 mM $MgCl_2$. (***I***) -2 $(iCy3)_2$ dimer at 20 mM NaCl and 0 mM $MgCl_2$. We note that the calculation for the sample shown in panel (*C*) exhibits two nearly stable minima within the error bar of ~$\pm$1%.

Statistical Uncertainty Analysis. We performed an analysis of the statistical uncertainty for the free parameters used in our kinetic network model for each of the $(iCy3)_2$ dimer-labeled ss-dsDNA

fork constructs at various salt conditions. This analysis is based on calculating the relative deviation of the global error function, $\Delta\chi^2_{tot}/\chi^2_{tot,0}$, from the optimized value, $\chi^2_{tot,0}$, as a function of the time constants and the mean visibility parameter uncertainties. The global error function dependence on the parameter uncertainties for the various constructs and salt conditions, shown in Fig. S6, demonstrate that the optimized values we obtained each correspond to a stable minimum. We assign upper bounds to the statistical uncertainties of these values to a 1% deviation of the total error function relative to the corresponding optimized value. From this analysis we obtain upper bounds of ~ $\pm$1% for the error bars of the free parameters listed in Table S1 and Table S5.

References

1. Phelps, C., B. Israels, D. Jose, M. C. Marsh, P. H. von Hippel, and A. H. Marcus. (2016) Using multi-order time correlation functions (TCFs) to elucidate biomolecular reaction pathways from microsecond single-molecule fluorescence experiments. *J. Phys. Chem. B*, **120**, 13003-13016.

2. Phelps, C., B. Israels, D. Jose, M. C. Marsh, P. H. von Hippel, and A. H. Marcus. (2017) Using microsecond single-molecule FRET to determine the assembly pathways of T4 ssDNA binding protein onto model DNA replication forks. *Proc. Nat. Acad. Sci. Plus USA*, **114**, E3612-E3621.

3. Israels, B., C. Albrecht, A. Dang, M. Barney, P. H. von Hippel, and A. H. Marcus. (2021) Sub-millisecond conformational transitions of single-stranded DNA lattices by photon correlation single-molecule FRET. *J. Phys. Chem. B*, **125**, 9426-9440.
