# Supplementary material for: Site-specific free energy surface parameters from single-molecule fluorescence measurements of exciton-coupled (iCy3)2 dimer probes positioned at DNA replication fork junctions
Source: Nucleic Acids Res. 2025 Feb 5;53(3):gkaf047. doi: 10.1093/nar/gkaf047 (PMC11795206; doi:10.1093/nar/gkaf047)
Supplement: gkaf047_Supplemental_Files [file gkaf047_supplemental_files.zip › Supplementary Tables.zip!/TableS1.docx]

**Table S1. Optimized parameters for the Gaussian components of the PS-SMF probability distribution functions (PDFs).**

| Sample | $A_{1}$ | $\bar{v}_{1}$ | $\sigma_{1}$ | $A_{2}$ | $\bar{v}_{2}$ | $\sigma_{2}$ | $A_{3}$ | $\bar{v}_{3}$ | $\sigma_{3}$ | $A_{4}$ | $\bar{v}_{4}$ | $\sigma_{4}$ | $A_{5}$ | $\bar{v}_{5}$ | $\sigma_{5}$ |
| --- | --- | --- | --- | --- | --- | --- | --- | --- | --- | --- | --- | --- | --- | --- | --- |
| +1, 300 mM NaCl | 0.08 | 0.003 | 0.037 | 0.59 | 0.11 | 0.049 | 4.84 | 0.15 | 0.075 | 0.02 | 0.32 | 0.072 | 0.12 | 0.39 | 0.054 |
| +1, 100 mM NaCl | 0.15 | 0.06 | 0.059 | 6.11 | 0.11 | 0.053 | 1.02 | 0.14 | 0.053 | 0.18 | 0.35 | 0.048 | - | - | - |
| +1, 20mM NaCl | 1.79 | 0.06 | 0.055 | 6.07 | 0.11 | 0.048 | 0.32 | 0.17 | 0.032 | 0.02 | 0.30 | 0.053 | - | - | - |
| +1, 20mM NaCl, 0 mM MgCl_2_ | 0.12 | 0.0001 | 0.019 | 5.31 | 0.12 | 0.062 | 1.01 | 0.14 | 0.060 | 0.19 | 0.37 | 0.028 | - | - | - |
| -1, 100mM NaCl | 0.22 | 0.0001 | 0.045 | 2.29 | 0.10 | 0.037 | 5.50 | 0.12 | 0.054 | 0.002 | 0.30 | 0.049 | - | - | - |
| -2, 300mM NaCl | 0.97 | 0.04 | 0.039 | 6.81 | 0.12 | 0.051 | 0.37 | 0.15 | 0.031 | 0.02 | 0.30 | 0.039 | - | - | - |
| -2, 100mM NaCl | 0.78 | 0.04 | 0.027 | 3.46 | 0.09 | 0.023 | 4.48 | 0.13 | 0.063 | 0.20 | 0.30 | 0.063 | - | - | - |
| -2, 20mM NaCl | 0.40 | 0.0001 | 0.038 | 6.71 | 0.12 | 0.054 | 0.39 | 0.17 | 0.044 | 0.07 | 0.30 | 0.02 | - | - | - |
| -2, 20mM NaCl, 0 mM MgCl_2_ | 1.43 | 0.06 | 0.034 | 6.32 | 0.11 | 0.037 | 1.31 | 0.19 | 0.045 | 1.09 | 0.24 | 0.045 | 0.01 | 0.32 | 0.046 |

Parameters defined in Eq. (5). Error bars for the mean visibilities are < 1% of reported values (see Statistical Uncertainty Analysis below). PDFs

were constructed using the integration period $T_{w}$ = 10 ms. All samples prepared in 10 mM Tris at pH 8.0 and 6 mM MgCl_2_,

unless otherwise specified.
