# Supplementary material for: Site-specific free energy surface parameters from single-molecule fluorescence measurements of exciton-coupled (iCy3)2 dimer probes positioned at DNA replication fork junctions
Source: Nucleic Acids Res. 2025 Feb 5;53(3):gkaf047. doi: 10.1093/nar/gkaf047 (PMC11795206; doi:10.1093/nar/gkaf047)
Supplement: gkaf047_Supplemental_Files [file gkaf047_supplemental_files.zip › Supplementary Tables.zip!/TableS2.docx]

**Table S2. Optimized values of the equilibrium probabilities and the visibility fluctuations.**

| Sample | $p_{1}^{eq}$ | $p_{2}^{eq}$ | $p_{3}^{eq}$ | $p_{4}^{eq}$ | $p_{5}^{eq}$ | $\delta\bar{v}_{1}$ | $\delta\bar{v}_{2}$ | ${\delta\bar{v}}_{3}$ | $\delta\bar{v}_{4}$ | $\delta\bar{v}_{5}$ | $\bar{v}$ |
| --- | --- | --- | --- | --- | --- | --- | --- | --- | --- | --- | --- |
| +1, 300 mM NaCl | 0.01 | 0.07 | 0.91 | 0.003 | 0.02 | -0.15 | -0.04 | 0.00 | 0.17 | 0.24 | 0.15 |
| +1, 100 mM NaCl | 0.02 | 0.82 | 0.14 | 0.02 | - | -0.06 | -0.01 | 0.02 | 0.23 | - | 0.12 |
| +1, 20mM NaCl | 0.25 | 0.74 | 0.02 | 0.002 | - | -0.04 | 0.01 | 0.07 | 0.20 | - | 0.10 |
| +1, 20mM NaCl, 0 mM MgCl_2_ | 0.01 | 0.82 | 0.15 | 0.01 | - | -0.12 | 0.00 | 0.02 | 0.25 | - | 0.12 |
| -1, 100mM NaCl | 0.02 | 0.22 | 0.76 | 0.0003 | - | -0.11 | -0.01 | 0.01 | 0.19 | - | 0.11 |
| -2, 300mM NaCl | 0.10 | 0.88 | 0.03 | 0.001 | - | -0.07 | 0.01 | 0.04 | 0.19 | - | 0.11 |
| -2, 100mM NaCl | 0.05 | 0.20 | 0.71 | 0.03 | - | -0.08 | -0.03 | 0.01 | 0.18 | - | 0.12 |
| -2, 20mM NaCl | 0.04 | 0.91 | 0.04 | 0.003 | - | -0.12 | 0.00 | 0.05 | 0.18 | - | 0.12 |
| -2, 20mM NaCl, 0 mM MgCl_2_ | 0.12 | 0.60 | 0.15 | 0.12 | 0.001 | -0.07 | -0.02 | 0.06 | 0.11 | 0.19 | 0.13 |

Equilibrium probabilities are calculated using the parameters listed in Table S1 and $p_{i}^{eq}=A_{i}\sigma_{i}\sqrt{2\pi}$.

Visibility fluctuations are calculated using the mean visibilities listed in Table S1 and $\delta\bar{v}_{i}=\bar{v}_{i}-\bar{v}$,

where $\bar{v}=\sum_{i=1}^{4} p_{i}^{eq}\bar{v}_{i}$.
