# Supplementary material for: Site-specific free energy surface parameters from single-molecule fluorescence measurements of exciton-coupled (iCy3)2 dimer probes positioned at DNA replication fork junctions
Source: Nucleic Acids Res. 2025 Feb 5;53(3):gkaf047. doi: 10.1093/nar/gkaf047 (PMC11795206; doi:10.1093/nar/gkaf047)
Supplement: gkaf047_Supplemental_Files [file gkaf047_supplemental_files.zip › Supplementary Tables.zip!/TableS3.docx]

**Table S3. Decomposition of largest pathway terms for the two-point TCFs [**${\bar{\boldsymbol{C}}}^{\left( \boldsymbol{2} \right)}$**function, Eq. (6)**

**of main text] for** $\boldsymbol{\tau}$ **= 250 μs.**

| +1, 300 mM NaCl, 6 mM MgCl_2_ | | | | | | | | | |
| --- | --- | --- | --- | --- | --- | --- | --- | --- | --- |
| Positive terms | | | | | Negative terms | | | | |
| $i$ | $j$ | $\delta\bar{v}_{i}\delta\bar{v}_{j}$ | $w_{ij}$ | $w_{ij}\delta\bar{v}_{i}\delta\bar{v}_{j}$ | $i$ | $j$ | $\delta\bar{v}_{i}\delta\bar{v}_{j}$ | $w_{ij}$ | $w_{ij}\delta\bar{v}_{i}\delta\bar{v}_{j}$ |
| 5 | 5 | 5.70 $\times$ 10^-2^ | 1.40 $\times$ 10^-2^ | 7.98 $\times$ 10^-4^ | 1 | 5 | -3.58 $\times$ 10^-2^ | 1.84 $\times$ 10^-3^ | -6.60 $\times$ 10^-5^ |
| 1 | 1 | 2.24 $\times$ 10^-2^ | 5.94 $\times$ 10^-3^ | 1.33 $\times$ 10^-4^ | 5 | 1 | -3.58 $\times$ 10^-2^ | 1.84 $\times$ 10^-3^ | -6.60 $\times$ 10^-5^ |
| 4 | 4 | 2.82 $\times$ 10^-2^ | 3.96 $\times$ 10^-3^ | 1.12 $\times$ 10^-4^ | 3 | 5 | -6.45 $\times$ 10^-4^ | 3.79 $\times$ 10^-4^ | -2.44 $\times$ 10^-7^ |
| 2 | 2 | 1.47 $\times$ 10^-3^ | 7.31 $\times$ 10^-2^ | 1.07 $\times$ 10^-4^ | 5 | 3 | -6.45 $\times$ 10^-4^ | 3.79 $\times$ 10^-4^ | -2.44 $\times$ 10^-7^ |
| 3 | 3 | 7.29 $\times$ 10^-6^ | 9.10 $\times$ 10^-1^ | 6.64 $\times$ 10^-6^ | 2 | 5 | -9.15 $\times$ 10^-3^ | 4.35 $\times$ 10^-6^ | -3.98 $\times$ 10^-8^ |
| 2 | 1 | 5.74 $\times$ 10^-3^ | 2.98 $\times$ 10^-5^ | 1.71 $\times$ 10^-7^ | 5 | 2 | -9.15 $\times$ 10^-3^ | 3.75 $\times$ 10^-6^ | -3.43 $\times$ 10^-8^ |
| 1 | 2 | 5.74 $\times$ 10^-3^ | 2.57 $\times$ 10^-5^ | 1.47 $\times$ 10^-7^ | 4 | 3 | -4.54 $\times$ 10^-4^ | 2.72 $\times$ 10^-6^ | -1.24 $\times$ 10^-9^ |
| 3 | 1 | 4.04 $\times$ 10^-4^ | 2.51 $\times$ 10^-5^ | 1.02 $\times$ 10^-8^ | 3 | 4 | -4.54 $\times$ 10^-4^ | 1.92 $\times$ 10^-6^ | -8.73 $\times$ 10^-10^ |

| +1, 100 mM NaCl, 6 mM MgCl_2_ | | | | | | | | | |
| --- | --- | --- | --- | --- | --- | --- | --- | --- | --- |
| Positive terms | | | | | Negative terms | | | | |
| $i$ | $j$ | $\delta\bar{v}_{i}\delta\bar{v}_{j}$ | $w_{ij}$ | $w_{ij}\delta\bar{v}_{i}\delta\bar{v}_{j}$ | $i$ | $j$ | $\delta\bar{v}_{i}\delta\bar{v}_{j}$ | $w_{ij}$ | $w_{ij}\delta\bar{v}_{i}\delta\bar{v}_{j}$ |
| 4 | 4 | 5.28 $\times$ 10^-2^ | 9.07 $\times$ 10^-3^ | 4.79 $\times$ 10^-4^ | 1 | 4 | -1.44 $\times$ 10^-2^ | 7.06 $\times$ 10^-4^ | -1.02 $\times$ 10^-5^ |
| 1 | 1 | 3.91 $\times$ 10^-3^ | 2.08 $\times$ 10^-2^ | 8.12 $\times$ 10^-5^ | 4 | 1 | -1.44 $\times$ 10^-2^ | 7.06 $\times$ 10^-4^ | -1.01 $\times$ 10^-5^ |
| 4 | 3 | 4.25 $\times$ 10^-3^ | 1.15 $\times$ 10^-2^ | 4.90 $\times$ 10^-5^ | 1 | 3 | -1.16 $\times$ 10^-3^ | 3.42 $\times$ 10^-4^ | -3.96 $\times$ 10^-7^ |
| 3 | 4 | 4.25 $\times$ 10^-3^ | 1.15 $\times$ 10^-2^ | 4.88 $\times$ 10^-5^ | 3 | 1 | -1.16 $\times$ 10^-3^ | 3.41 $\times$ 10^-4^ | -3.95 $\times$ 10^-7^ |
| 2 | 2 | 5.62 $\times$ 10^-5^ | 8.19 $\times$ 10^-1^ | 4.60 $\times$ 10^-5^ | 2 | 4 | -1.72 $\times$ 10^-3^ | 7.67 $\times$ 10^-5^ | -1.32 $\times$ 10^-7^ |
| 3 | 3 | 3.43 $\times$ 10^-4^ | 1.26 $\times$ 10^-1^ | 4.32 $\times$ 10^-5^ | 4 | 2 | -1.72 $\times$ 10^-3^ | 4.01 $\times$ 10^-5^ | -6.91 $\times$ 10^-8^ |
| 2 | 1 | 4.69 $\times$ 10^-4^ | 2.21 $\times$ 10^-6^ | 1.04 $\times$ 10^-9^ | 3 | 2 | -1.39 $\times$ 10^-4^ | 7.74 $\times$ 10^-5^ | -1.08 $\times$ 10^-8^ |
| 1 | 2 | 4.69 $\times$ 10^-4^ | 1.12 $\times$ 10^-6^ | 5.28 $\times$ 10^-10^ | 2 | 3 | -1.39 $\times$ 10^-4^ | 3.96 $\times$ 10^-5^ | -5.50 $\times$ 10^-9^ |

| +1, 20 mM NaCl, 6 mM MgCl_2_ | | | | | | | | | |
| --- | --- | --- | --- | --- | --- | --- | --- | --- | --- |
| Positive terms | | | | | Negative terms | | | | |
| $i$ | $j$ | $\delta\bar{v}_{i}\delta\bar{v}_{j}$ | $w_{ij}$ | $w_{ij}\delta\bar{v}_{i}\delta\bar{v}_{j}$ | $i$ | $j$ | $\delta\bar{v}_{i}\delta\bar{v}_{j}$ | $w_{ij}$ | $w_{ij}\delta\bar{v}_{i}\delta\bar{v}_{j}$ |
| 1 | 1 | 1.76 $\times$ 10^-3^ | 1.64 $\times$ 10^-3^ | 2.89 $\times$ 10^-4^ | 1 | 2 | -3.38 $\times$ 10^-4^ | 8.48 $\times$ 10^-2^ | -2.87 $\times$ 10^-5^ |
| 4 | 4 | 3.53 $\times$ 10^-2^ | 2.66 $\times$ 10^-3^ | 9.42 $\times$ 10^-5^ | 2 | 1 | -3.38 $\times$ 10^-4^ | 8.48 $\times$ 10^-2^ | -2.87 $\times$ 10^-5^ |
| 3 | 3 | 3.52 $\times$ 10^-3^ | 2.56 $\times$ 10^-2^ | 9.02 $\times$ 10^-5^ | 4 | 1 | -7.89 $\times$ 10^-3^ | 3.84 $\times$ 10^-5^ | -3.03 $\times$ 10^-7^ |
| 2 | 2 | 6.50 $\times$ 10^-5^ | 6.60 $\times$ 10^-1^ | 4.28 $\times$ 10^-5^ | 1 | 4 | -7.89 $\times$ 10^-3^ | 3.84 $\times$ 10^-4^ | -3.03 $\times$ 10^-7^ |
| 4 | 2 | 1.51 $\times$ 10^-3^ | 8.88 $\times$ 10^-6^ | 1.34 $\times$ 10^-8^ | 3 | 1 | -2.49 $\times$ 10^-3^ | 6.24 $\times$ 10^-7^ | -1.56 $\times$ 10^-9^ |
| 2 | 4 | 1.51 $\times$ 10^-3^ | 8.88 $\times$ 10^-6^ | 1.34 $\times$ 10^-8^ | 1 | 3 | -2.49 $\times$ 10^-3^ | 9.72 $\times$ 10^-8^ | -2.42 $\times$ 10^-10^ |
| 3 | 2 | 4.78 $\times$ 10^-5^ | 9.33 $\times$ 10^-6^ | 4.47 $\times$ 10^-9^ |  |  |  |  |  |
| 2 | 3 | 4.78 $\times$ 10^-4^ | 1.45 $\times$ 10^-6^ | 6.95 $\times$ 10^-10^ |  |  |  |  |  |

| +1, 20 mM NaCl, 0 mM MgCl_2_ | | | | | | | | | |
| --- | --- | --- | --- | --- | --- | --- | --- | --- | --- |
| Positive terms | | | | | Negative terms | | | | |
| $i$ | $j$ | $\delta\bar{v}_{i}\delta\bar{v}_{j}$ | $w_{ij}$ | $w_{ij}\delta\bar{v}_{i}\delta\bar{v}_{j}$ | $i$ | $j$ | $\delta\bar{v}_{i}\delta\bar{v}_{j}$ | $w_{ij}$ | $w_{ij}\delta\bar{v}_{i}\delta\bar{v}_{j}$ |
| 4 | 4 | 5.80 $\times$ 10^-2^ | 7.17 $\times$ 10^-3^ | 4.16 $\times$ 10^-4^ | 1 | 3 | -1.75 $\times$ 10^-3^ | 3.03 $\times$ 10^-4^ | -5.30 $\times$ 10^-7^ |
| 1 | 1 | 1.64 $\times$ 10^-2^ | 5.61 $\times$ 10^-3^ | 9.27 $\times$ 10^-5^ | 3 | 1 | -1.75 $\times$ 10^-3^ | 3.03 $\times$ 10^-4^ | -5.30 $\times$ 10^-7^ |
| 3 | 3 | 1.85 $\times$ 10^-4^ | 1.48 $\times$ 10^-1^ | 2.74 $\times$ 10^-5^ | 2 | 4 | -1.36 $\times$ 10^-3^ | 2.09 $\times$ 10^-4^ | -2.83 $\times$ 10^-7^ |
| 2 | 2 | 3.18 $\times$ 10^-5^ | 8.25 $\times$ 10^-1^ | 2.62 $\times$ 10^-5^ | 4 | 1 | -3.10 $\times$ 10^-2^ | 7.43 $\times$ 10^-6^ | -2.30 $\times$ 10^-7^ |
| 4 | 3 | 3.28 $\times$ 10^-3^ | 6.54 $\times$ 10^-3^ | 2.14 $\times$ 10^-5^ | 1 | 4 | -3.10 $\times$ 10^-2^ | 7.21 $\times$ 10^-6^ | -2.23 $\times$ 10^-7^ |
| 3 | 4 | 3.28 $\times$ 10^-3^ | 6.35 $\times$ 10^-3^ | 2.08 $\times$ 10^-5^ | 3 | 2 | -7.67 $\times$ 10^-5^ | 3.49 $\times$ 10^-4^ | -2.68 $\times$ 10^-8^ |
| 1 | 2 | 7.24 $\times$ 10^-4^ | 3.55 $\times$ 10^-7^ | 2.57 $\times$ 10^-10^ | 4 | 2 | -1.35 $\times$ 10^-3^ | 1.53 $\times$ 10^-5^ | -2.07 $\times$ 10^-8^ |
| 2 | 1 | 7.24 $\times$ 10^-4^ | 1.36 $\times$ 10^-7^ | 9.86 $\times$ 10^-11^ | 2 | 3 | -7.67 $\times$ 10^-5^ | 1.56 $\times$ 10^-4^ | -1.19 $\times$ 10^-8^ |

| -1, 100 mM NaCl, 6 mM MgCl_2_ | | | | | | | | | |
| --- | --- | --- | --- | --- | --- | --- | --- | --- | --- |
| Positive terms | | | | | Negative terms | | | | |
| $i$ | $j$ | $\delta\bar{v}_{i}\delta\bar{v}_{j}$ | $w_{ij}$ | $w_{ij}\delta\bar{v}_{i}\delta\bar{v}_{j}$ | $i$ | $j$ | $\delta\bar{v}_{i}\delta\bar{v}_{j}$ | $w_{ij}$ | $w_{ij}\delta\bar{v}_{i}\delta\bar{v}_{j}$ |
| 1 | 1 | 1.31 $\times$ 10^-2^ | 2.06 $\times$ 10^-2^ | 2.70 $\times$ 10^-4^ | 3 | 1 | -8.88 $\times$ 10^-4^ | 4.08 $\times$ 10^-3^ | -3.63 $\times$ 10^-6^ |
| 3 | 3 | 6.02 $\times$ 10^-5^ | 7.52 $\times$ 10^-1^ | 4.53 $\times$ 10^-5^ | 1 | 3 | -8.88 $\times$ 10^-4^ | 3.97 $\times$ 10^-3^ | -3.53 $\times$ 10^-6^ |
| 2 | 2 | 1.85 $\times$ 10^-4^ | 2.16 $\times$ 10^-1^ | 4.00 $\times$ 10^-5^ | 2 | 3 | -1.06 $\times$ 10^-4^ | 2.36 $\times$ 10^-4^ | -2.49 $\times$ 10^-8^ |
| 4 | 4 | 3.44 $\times$ 10^-2^ | 2.75 $\times$ 10^-4^ | 9.47 $\times$ 10^-6^ | 4 | 1 | -2.12 $\times$ 10^-2^ | 7.44 $\times$ 10^-7^ | -1.58 $\times$ 10^-8^ |
| 1 | 2 | 1.56 $\times$ 10^-3^ | 1.35 $\times$ 10^-3^ | 2.10 $\times$ 10^-6^ | 1 | 4 | -2.12 $\times$ 10^-2^ | 7.44 $\times$ 10^-7^ | -1.58 $\times$ 10^-8^ |
| 2 | 1 | 1.56 $\times$ 10^-3^ | 1.24 $\times$ 10^-3^ | 1.93 $\times$ 10^-6^ | 3 | 2 | -1.06 $\times$ 10^-4^ | 1.26 $\times$ 10^-4^ | -1.33 $\times$ 10^-8^ |
| 3 | 4 | 1.44 $\times$ 10^-3^ | 6.83 $\times$ 10^-8^ | 9.84 $\times$ 10^-11^ | 4 | 2 | -2.52 $\times$ 10^-3^ | 2.26 $\times$ 10^-8^ | -5.70 $\times$ 10^-11^ |
| 4 | 3 | 1.44 $\times$ 10^-3^ | 6.65 $\times$ 10^-8^ | 9.57 $\times$ 10^-11^ | 2 | 4 | -2.52 $\times$ 10^-3^ | 2.07 $\times$ 10^-8^ | -5.24 $\times$ 10^-11^ |

| -2, 300 mM NaCl, 6 mM MgCl_2_ | | | | | | | | | |
| --- | --- | --- | --- | --- | --- | --- | --- | --- | --- |
| Positive terms | | | | | Negative terms | | | | |
| $i$ | $j$ | $\delta\bar{v}_{i}\delta\bar{v}_{j}$ | $w_{ij}$ | $w_{ij}\delta\bar{v}_{i}\delta\bar{v}_{j}$ | $i$ | $j$ | $\delta\bar{v}_{i}\delta\bar{v}_{j}$ | $w_{ij}$ | $w_{ij}\delta\bar{v}_{i}\delta\bar{v}_{j}$ |
| 1 | 1 | 5.52 $\times$ 10^-3^ | 5.27 $\times$ 10^-2^ | 2.91 $\times$ 10^-4^ | 1 | 2 | -3.89 $\times$ 10^-4^ | 4.27 $\times$ 10^-2^ | -1.66 $\times$ 10^-5^ |
| 4 | 4 | 3.44 $\times$ 10^-2^ | 1.88 $\times$ 10^-3^ | 6.46 $\times$ 10^-5^ | 2 | 1 | -3.89 $\times$ 10^-4^ | 4.27 $\times$ 10^-2^ | -1.66 $\times$ 10^-5^ |
| 3 | 3 | 1.74 $\times$ 10^-3^ | 2.88 $\times$ 10^-2^ | 5.02 $\times$ 10^-5^ | 4 | 1 | -1.38 $\times$ 10^-2^ | 3.28 $\times$ 10^-5^ | -4.52 $\times$ 10^-7^ |
| 2 | 2 | 2.74 $\times$ 10^-5^ | 8.39 $\times$ 10^-1^ | 2.30 $\times$ 10^-5^ | 1 | 4 | -1.38 $\times$ 10^-2^ | 3.28 $\times$ 10^-5^ | -4.52 $\times$ 10^-7^ |
| 4 | 2 | 9.71 $\times$ 10^-4^ | 1.09 $\times$ 10^-5^ | 1.06 $\times$ 10^-8^ | 3 | 1 | -3.10 $\times$ 10^-3^ | 3.74 $\times$ 10^-7^ | -1.16 $\times$ 10^-9^ |
| 2 | 4 | 9.71 $\times$ 10^-4^ | 1.09 $\times$ 10^-5^ | 1.06 $\times$ 10^-8^ | 1 | 3 | -3.10 $\times$ 10^-3^ | 2.72 $\times$ 10^-7^ | -8.46 $\times$ 10^-10^ |
| 3 | 2 | 2.18 $\times$ 10^-4^ | 1.35 $\times$ 10^-5^ | 2.95 $\times$ 10^-9^ |  |  |  |  |  |
| 2 | 3 | 2.18 $\times$ 10^-4^ | 9.84 $\times$ 10^-6^ | 2.15 $\times$ 10^-9^ |  |  |  |  |  |

| -2, 100 mM NaCl, 6 mM MgCl_2_ | | | | | | | | | |
| --- | --- | --- | --- | --- | --- | --- | --- | --- | --- |
| Positive terms | | | | | Negative terms | | | | |
| $i$ | $j$ | $\delta\bar{v}_{i}\delta\bar{v}_{j}$ | $w_{ij}$ | $w_{ij}\delta\bar{v}_{i}\delta\bar{v}_{j}$ | $i$ | $j$ | $\delta\bar{v}_{i}\delta\bar{v}_{j}$ | $w_{ij}$ | $w_{ij}\delta\bar{v}_{i}\delta\bar{v}_{j}$ |
| 4 | 4 | 3.02 $\times$ 10^-2^ | 1.98 $\times$ 10^-2^ | 5.98 $\times$ 10^-4^ | 4 | 1 | -1.33 $\times$ 10^-2^ | 1.15 $\times$ 10^-2^ | -1.54 $\times$ 10^-5^ |
| 1 | 1 | 5.86 $\times$ 10^-3^ | 3.74 $\times$ 10^-2^ | 2.19 $\times$ 10^-4^ | 1 | 4 | -1.33 $\times$ 10^-2^ | 1.15 $\times$ 10^-2^ | -1.54 $\times$ 10^-5^ |
| 2 | 2 | 9.22 $\times$ 10^-4^ | 1.97 $\times$ 10^-1^ | 1.82 $\times$ 10^-4^ | 2 | 4 | -5.28 $\times$ 10^-3^ | 7.63 $\times$ 10^-4^ | -4.03 $\times$ 10^-6^ |
| 3 | 3 | 4.41 $\times$ 10^-5^ | 7.10 $\times$ 10^-1^ | 3.13 $\times$ 10^-5^ | 4 | 2 | -5.28 $\times$ 10^-3^ | 7.62 $\times$ 10^-4^ | -4.02 $\times$ 10^-6^ |
| 1 | 2 | 2.32 $\times$ 10^-3^ | 4.95 $\times$ 10^-3^ | 1.15 $\times$ 10^-5^ | 2 | 3 | -2.02 $\times$ 10^-4^ | 4.57 $\times$ 10^-4^ | -9.22 $\times$ 10^-8^ |
| 2 | 1 | 2.32 $\times$ 10^-3^ | 4.95 $\times$ 10^-3^ | 1.15 $\times$ 10^-5^ | 3 | 2 | -2.02 $\times$ 10^-4^ | 4.57 $\times$ 10^-4^ | -9.22 $\times$ 10^-8^ |
| 3 | 4 | 1.15 $\times$ 10^-3^ | 6.32 $\times$ 10^-7^ | 7.30 $\times$ 10^-10^ | 1 | 3 | -5.08 $\times$ 10^-4^ | 6.04 $\times$ 10^-6^ | -3.07 $\times$ 10^-9^ |
| 4 | 3 | 1.15 $\times$ 10^-3^ | 6.30 $\times$ 10^-7^ | 7.28 $\times$ 10^-10^ | 3 | 1 | -5.08 $\times$ 10^-4^ | 6.04 $\times$ 10^-6^ | -3.07 $\times$ 10^-9^ |

| -2, 20 mM NaCl, 6 mM MgCl_2_ | | | | | | | | | |
| --- | --- | --- | --- | --- | --- | --- | --- | --- | --- |
| Positive terms | | | | | Negative terms | | | | |
| $i$ | $j$ | $\delta\bar{v}_{i}\delta\bar{v}_{j}$ | $w_{ij}$ | $w_{ij}\delta\bar{v}_{i}\delta\bar{v}_{j}$ | $i$ | $j$ | $\delta\bar{v}_{i}\delta\bar{v}_{j}$ | $w_{ij}$ | $w_{ij}\delta\bar{v}_{i}\delta\bar{v}_{j}$ |
| 1 | 1 | 1.36 $\times$ 10^-2^ | 2.51 $\times$ 10^-2^ | 3.40 $\times$ 10^-4^ | 1 | 2 | -1.95 $\times$ 10^-4^ | 1.41 $\times$ 10^-2^ | -2.75 $\times$ 10^-6^ |
| 3 | 3 | 2.62 $\times$ 10^-3^ | 4.38 $\times$ 10^-2^ | 1.15 $\times$ 10^-4^ | 2 | 1 | -1.95 $\times$ 10^-4^ | 1.41 $\times$ 10^-2^ | -2.74 $\times$ 10^-6^ |
| 4 | 4 | 3.35 $\times$ 10^-2^ | 3.36 $\times$ 10^-3^ | 1.12 $\times$ 10^-4^ | 4 | 1 | -2.13 $\times$ 10^-3^ | 4.36 $\times$ 10^-5^ | -9.29 $\times$ 10^-7^ |
| 2 | 2 | 2.80 $\times$ 10^-6^ | 9.01 $\times$ 10^-1^ | 2.52 $\times$ 10^-6^ | 1 | 4 | -2.13 $\times$ 10^-3^ | 4.36 $\times$ 10^-5^ | -9.29 $\times$ 10^-7^ |
| 4 | 2 | 3.06 $\times$ 10^-4^ | 1.05 $\times$ 10^-5^ | 3.22 $\times$ 10^-9^ | 3 | 1 | -5.96 $\times$ 10^-3^ | 1.79 $\times$ 10^-5^ | -1.07 $\times$ 10^-7^ |
| 2 | 4 | 3.06 $\times$ 10^-4^ | 1.05 $\times$ 10^-5^ | 3.22 $\times$ 10^-9^ | 1 | 3 | -5.96 $\times$ 10^-3^ | 3.76 $\times$ 10^-7^ | -2.24 $\times$ 10^-9^ |
| 2 | 3 | 8.56 $\times$ 10^-5^ | 2.68 $\times$ 10^-5^ | 2.30 $\times$ 10^-9^ |  |  |  |  |  |
| 3 | 2 | 8.56 $\times$ 10^-5^ | 1.02 $\times$ 10^-5^ | 8.70 $\times$ 10^-10^ |  |  |  |  |  |

| -2, 20 mM NaCl, 0 mM MgCl_2_ | | | | | | | | | |
| --- | --- | --- | --- | --- | --- | --- | --- | --- | --- |
| Positive terms | | | | | Negative terms | | | | |
| $i$ | $j$ | $\delta\bar{v}_{i}\delta\bar{v}_{j}$ | $w_{ij}$ | $w_{ij}\delta\bar{v}_{i}\delta\bar{v}_{j}$ | $i$ | $j$ | $\delta\bar{v}_{i}\delta\bar{v}_{j}$ | $w_{ij}$ | $w_{ij}\delta\bar{v}_{i}\delta\bar{v}_{j}$ |
| 4 | 4 | 1.24 $\times$ 10^-2^ | 6.11 $\times$ 10^-2^ | 7.61 $\times$ 10^-4^ | 4 | 1 | -8.86 $\times$ 10^-3^ | 6.11 $\times$ 10^-2^ | -5.41 $\times$ 10^-4^ |
| 1 | 1 | 6.30 $\times$ 10^-3^ | 6.11 $\times$ 10^-2^ | 3.85 $\times$ 10^-4^ | 1 | 4 | -8.86 $\times$ 10^-3^ | 6.11 $\times$ 10^-2^ | -5.41 $\times$ 10^-4^ |
| 3 | 3 | 2.79 $\times$ 10^-3^ | 1.20 $\times$ 10^-1^ | 3.35 $\times$ 10^-4^ | 2 | 3 | -1.06 $\times$ 10^-3^ | 2.81 $\times$ 10^-2^ | -2.98 $\times$ 10^-5^ |
| 2 | 2 | 4.05 $\times$ 10^-4^ | 5.73 $\times$ 10^-1^ | 2.32 $\times$ 10^-4^ | 3 | 2 | -1.06 $\times$ 10^-3^ | 2.81 $\times$ 10^-2^ | -2.98 $\times$ 10^-5^ |
| 5 | 5 | 3.29 $\times$ 10^-2^ | 5.90 $\times$ 10^-4^ | 1.94 $\times$ 10^-5^ | 1 | 3 | -4.19 $\times$ 10^-3^ | 1.32 $\times$ 10^-3^ | -5.53 $\times$ 10^-6^ |
| 3 | 4 | 5.89 $\times$ 10^-3^ | 1.32 $\times$ 10^-3^ | 7.76 $\times$ 10^-6^ | 3 | 1 | -4.19 $\times$ 10^-3^ | 1.32 $\times$ 10^-3^ | -5.53 $\times$ 10^-6^ |
| 4 | 3 | 5.89 $\times$ 10^-3^ | 1.32 $\times$ 10^-3^ | 7.76 $\times$ 10^-6^ | 2 | 4 | -2.24 $\times$ 10^-3^ | 1.44 $\times$ 10^-4^ | -3.23 $\times$ 10^-7^ |
| 1 | 2 | 1. 60 $\times$ 10^-3^ | 1.44 $\times$ 10^-4^ | 2.30 $\times$ 10^-7^ | 4 | 2 | -2.24 $\times$ 10^-3^ | 1.44 $\times$ 10^-4^ | -3.23 $\times$ 10^-7^ |
