# Supplementary material for: Site-specific free energy surface parameters from single-molecule fluorescence measurements of exciton-coupled (iCy3)2 dimer probes positioned at DNA replication fork junctions
Source: Nucleic Acids Res. 2025 Feb 5;53(3):gkaf047. doi: 10.1093/nar/gkaf047 (PMC11795206; doi:10.1093/nar/gkaf047)
Supplement: gkaf047_Supplemental_Files [file gkaf047_supplemental_files.zip › Supplementary Tables.zip!/TableS4.docx]

**Table S4. Decomposition of largest pathway terms for the three-point TCFs [**${\bar{\boldsymbol{C}}}^{\left( \boldsymbol{3} \right)}$**function, Eq. (7) of main text] for** $\boldsymbol{\tau}_{\boldsymbol{1}}\boldsymbol{=}\boldsymbol{\tau}_{\boldsymbol{2}}$ **= 10 ms.**

| +1, 300 mM NaCl, 6 mM MgCl_2_ | | | | | | | | | | | |
| --- | --- | --- | --- | --- | --- | --- | --- | --- | --- | --- | --- |
| Positive terms | | | | | | Negative terms | | | | | |
| $i$ | $j$ | $k$ | $\delta\bar{v}_{i}\delta\bar{v}_{j}\delta\bar{v}_{k}$ | $w_{ijk}$ | $w_{ijk}\delta\bar{v}_{i}\delta\bar{v}_{j}\delta\bar{v}_{k}$ | $i$ | $j$ | $k$ | $\delta\bar{v}_{i}\delta\bar{v}_{j}\delta\bar{v}_{k}$ | $w_{ijk}$ | $w_{ijk}\delta\bar{v}_{i}\delta\bar{v}_{j}\delta\bar{v}_{k}$ |
| 5 | 5 | 5 | 1.36 $\times$ 10^-2^ | 1.66 $\times$ 10^-3^ | 2.66 $\times$ 10^-5^ | 5 | 1 | 5 | -8.54 $\times$ 10^-3^ | 8.86 $\times$ 10^-4^ | -7.56 $\times$ 10^-6^ |
| 4 | 4 | 4 | 4.73 $\times$ 10^-3^ | 3.78 $\times$ 10^-3^ | 1.79 $\times$ 10^-5^ | 1 | 5 | 5 | -8.54 $\times$ 10^-3^ | 8.44 $\times$ 10^-4^ | -7.21 $\times$ 10^-6^ |
| 1 | 1 | 5 | 5.36 $\times$ 10^-3^ | 4.49 $\times$ 10^-4^ | 2.40 $\times$ 10^-6^ | 5 | 5 | 1 | -8.54 $\times$ 10^-3^ | 8.44 $\times$ 10^-4^ | -7.21 $\times$ 10^-6^ |
| 5 | 1 | 1 | 5.36 $\times$ 10^-3^ | 4.49 $\times$ 10^-4^ | 2.40 $\times$ 10^-6^ | 2 | 2 | 2 | -5.64 $\times$ 10^-5^ | 7.06 $\times$ 10^-2^ | -3.98 $\times$ 10^-6^ |
| 1 | 5 | 1 | 5.36 $\times$ 10^-3^ | 4.28 $\times$ 10^-4^ | 2.29 $\times$ 10^-6^ | 1 | 1 | 1 | -3.36 $\times$ 10^-3^ | 2.28 $\times$ 10^-4^ | -7.65 $\times$ 10^-7^ |
| 2 | 2 | 5 | 3.51 $\times$ 10^-4^ | 6.03 $\times$ 10^-4^ | 2.12 $\times$ 10^-7^ | 2 | 5 | 5 | -2.19 $\times$ 10^-3^ | 1.97 $\times$ 10^-4^ | -4.30 $\times$ 10^-7^ |
| 5 | 2 | 2 | 3.51 $\times$ 10^-4^ | 5.18 $\times$ 10^-4^ | 1.82 $\times$ 10^-7^ | 3 | 5 | 5 | -1.56 $\times$ 10^-4^ | 2.52 $\times$ 10^-3^ | -3.93 $\times$ 10^-7^ |
| 2 | 1 | 5 | 1.37 $\times$ 10^-3^ | 1.30 $\times$ 10^-4^ | 1.78 $\times$ 10^-7^ | 5 | 5 | 3 | -1.56 $\times$ 10^-4^ | 2.52 $\times$ 10^-3^ | -3.93 $\times$ 10^-7^ |

| +1, 100 mM NaCl, 6 mM MgCl_2_ | | | | | | | | | | | |
| --- | --- | --- | --- | --- | --- | --- | --- | --- | --- | --- | --- |
| Positive terms | | | | | | Negative terms | | | | | |
| $i$ | $j$ | $k$ | $\delta\bar{v}_{i}\delta\bar{v}_{j}\delta\bar{v}_{k}$ | $w_{ijk}$ | $w_{ijk}\delta\bar{v}_{i}\delta\bar{v}_{j}\delta\bar{v}_{k}$ | $i$ | $j$ | $k$ | $\delta\bar{v}_{i}\delta\bar{v}_{j}\delta\bar{v}_{k}$ | $w_{ijk}$ | $w_{ijk}\delta\bar{v}_{i}\delta\bar{v}_{j}\delta\bar{v}_{k}$ |
| 4 | 4 | 4 | 1.21 $\times$ 10^-2^ | 2.80 $\times$ 10^-4^ | 3.40 $\times$ 10^-6^ | 1 | 4 | 4 | -3.30 $\times$ 10^-3^ | 2.68 $\times$ 10^-4^ | -8.83 $\times$ 10^-7^ |
| 4 | 3 | 4 | 9.77 $\times$ 10^-4^ | 1.84 $\times$ 10^-3^ | 1.80 $\times$ 10^-6^ | 4 | 4 | 1 | -3.30 $\times$ 10^-3^ | 2.68 $\times$ 10^-4^ | -8.83 $\times$ 10^-7^ |
| 4 | 4 | 3 | 9.77 $\times$ 10^-4^ | 1.83 $\times$ 10^-3^ | 1.79 $\times$ 10^-6^ | 4 | 1 | 4 | -3.30 $\times$ 10^-3^ | 2.50 $\times$ 10^-4^ | -8.24 $\times$ 10^-7^ |
| 3 | 4 | 4 | 9.77 $\times$ 10^-4^ | 1.82 $\times$ 10^-3^ | 1.78 $\times$ 10^-6^ | 1 | 4 | 3 | -2.66 $\times$ 10^-4^ | 1.74 $\times$ 10^-3^ | -4.64 $\times$ 10^-7^ |
| 4 | 3 | 3 | 7.88 $\times$ 10^-5^ | 1.20 $\times$ 10^-2^ | 9.47 $\times$ 10^-7^ | 3 | 4 | 1 | -2.66 $\times$ 10^-4^ | 1.74 $\times$ 10^-3^ | -4.63 $\times$ 10^-7^ |
| 3 | 3 | 4 | 7.88 $\times$ 10^-5^ | 1.20 $\times$ 10^-2^ | 9.44 $\times$ 10^-7^ | 1 | 3 | 4 | -2.66 $\times$ 10^-4^ | 1.66 $\times$ 10^-3^ | -4.41 $\times$ 10^-7^ |
| 3 | 4 | 3 | 7.88 $\times$ 10^-5^ | 1.19 $\times$ 10^-2^ | 9.36 $\times$ 10^-7^ | 4 | 3 | 1 | -2.66 $\times$ 10^-4^ | 1.66 $\times$ 10^-3^ | -4.41 $\times$ 10^-7^ |
| 3 | 3 | 3 | 6.34 $\times$ 10^-6^ | 7.84 $\times$ 10^-2^ | 4.898 $\times$ 10^-7^ | 4 | 1 | 3 | -2.66 $\times$ 10^-4^ | 1.54 $\times$ 10^-3^ | -4.09 $\times$ 10^-7^ |

| +1, 20 mM NaCl, 6 mM MgCl_2_ | | | | | | | | | | | |
| --- | --- | --- | --- | --- | --- | --- | --- | --- | --- | --- | --- |
| Positive terms | | | | | | Negative terms | | | | | |
| $i$ | $j$ | $k$ | $\delta\bar{v}_{i}\delta\bar{v}_{j}\delta\bar{v}_{k}$ | $w_{ijk}$ | $w_{ijk}\delta\bar{v}_{i}\delta\bar{v}_{j}\delta\bar{v}_{k}$ | $i$ | $j$ | $k$ | $\delta\bar{v}_{i}\delta\bar{v}_{j}\delta\bar{v}_{k}$ | $w_{ijk}$ | $w_{ijk}\delta\bar{v}_{i}\delta\bar{v}_{j}\delta\bar{v}_{k}$ |
| 3 | 3 | 3 | 2.09 $\times$ 10^-4^ | 2.51 $\times$ 10^-2^ | 5.24 $\times$ 10^-6^ | 1 | 1 | 1 | -7.41 $\times$ 10^-5^ | 1.56 $\times$ 10^-2^ | -1.16 $\times$ 10^-6^ |
| 4 | 4 | 4 | 6.64 $\times$ 10^-3^ | 6.65 $\times$ 10^-4^ | 4.41 $\times$ 10^-6^ | 1 | 2 | 2 | -2.69 $\times$ 10^-6^ | 1.39 $\times$ 10^-1^ | -3.75 $\times$ 10^-7^ |
| 1 | 2 | 1 | 1.41 $\times$ 10^-5^ | 4.67 $\times$ 10^-2^ | 6.59 $\times$ 10^-7^ | 2 | 2 | 1 | -2.69 $\times$ 10^-6^ | 1.39 $\times$ 10^-1^ | -3.75 $\times$ 10^-7^ |
| 1 | 1 | 2 | 1.41 $\times$ 10^-5^ | 4.67 $\times$ 10^-2^ | 6.59 $\times$ 10^-7^ | 2 | 1 | 2 | -2.69 $\times$ 10^-6^ | 1.39 $\times$ 10^-1^ | -3.75 $\times$ 10^-7^ |
| 2 | 1 | 1 | 1.41 $\times$ 10^-5^ | 4.67 $\times$ 10^-2^ | 6.59 $\times$ 10^-7^ | 4 | 4 | 1 | -1.48 $\times$ 10^-3^ | 1.85 $\times$ 10^-4^ | -2.75 $\times$ 10^-7^ |
| 2 | 2 | 2 | 5.12 $\times$ 10^-7^ | 4.16 $\times$ 10^-1^ | 2.13 $\times$ 10^-7^ | 1 | 4 | 4 | -1.48 $\times$ 10^-3^ | 1.85 $\times$ 10^-4^ | -2.75 $\times$ 10^-7^ |
| 4 | 4 | 2 | 2.83 $\times$ 10^-4^ | 4.93 $\times$ 10^-4^ | 1.39 $\times$ 10^-7^ | 4 | 1 | 2 | -6.32 $\times$ 10^-5^ | 2.79 $\times$ 10^-4^ | -1.76 $\times$ 10^-8^ |
| 2 | 4 | 4 | 2.83 $\times$ 10^-4^ | 4.93 $\times$ 10^-4^ | 1.39 $\times$ 10^-7^ | 2 | 1 | 4 | -6.32 $\times$ 10^-5^ | 2.79 $\times$ 10^-4^ | -1.76 $\times$ 10^-8^ |

| +1, 20 mM NaCl, 0 mM MgCl_2_ | | | | | | | | | | | |
| --- | --- | --- | --- | --- | --- | --- | --- | --- | --- | --- | --- |
| Positive terms | | | | | | Negative terms | | | | | |
| $i$ | $j$ | $k$ | $\delta\bar{v}_{i}\delta\bar{v}_{j}\delta\bar{v}_{k}$ | $w_{ijk}$ | $w_{ijk}\delta\bar{v}_{i}\delta\bar{v}_{j}\delta\bar{v}_{k}$ | $i$ | $j$ | $k$ | $\delta\bar{v}_{i}\delta\bar{v}_{j}\delta\bar{v}_{k}$ | $w_{ijk}$ | $w_{ijk}\delta\bar{v}_{i}\delta\bar{v}_{j}\delta\bar{v}_{k}$ |
| 4 | 4 | 4 | 1.40 $\times$ 10^-2^ | 6.93 $\times$ 10^-5^ | 9.69 $\times$ 10^-7^ | 1 | 1 | 1 | -2.12 $\times$ 10^-3^ | 1.11 $\times$ 10^-4^ | -2.36 $\times$ 10^-7^ |
| 4 | 4 | 3 | 7.90 $\times$ 10^-4^ | 8.02 $\times$ 10^-4^ | 6.34 $\times$ 10^-7^ | 4 | 4 | 1 | -7.46 $\times$ 10^-3^ | 2.78 $\times$ 10^-5^ | -2.07 $\times$ 10^-7^ |
| 4 | 3 | 4 | 7.90 $\times$ 10^-4^ | 8.00 $\times$ 10^-4^ | 6.32 $\times$ 10^-7^ | 1 | 4 | 4 | -7.46 $\times$ 10^-3^ | 2.70 $\times$ 10^-5^ | -2.01 $\times$ 10^-7^ |
| 3 | 4 | 4 | 7.90 $\times$ 10^-4^ | 7.78 $\times$ 10^-4^ | 6.15 $\times$ 10^-7^ | 4 | 1 | 4 | -7.46 $\times$ 10^-3^ | 2.51 $\times$ 10^-5^ | -1.87 $\times$ 10^-7^ |
| 4 | 3 | 3 | 4.47 $\times$ 10^-5^ | 9.25 $\times$ 10^-3^ | 4.13 $\times$ 10^-7^ | 2 | 2 | 2 | -1.79 $\times$ 10^-7^ | 7.98 $\times$ 10^-1^ | -1.43 $\times$ 10^-7^ |
| 3 | 4 | 3 | 4.47 $\times$ 10^-5^ | 9.00 $\times$ 10^-3^ | 4.02 $\times$ 10^-7^ | 4 | 3 | 1 | -4.22 $\times$ 10^-4^ | 3.24 $\times$ 10^-4^ | -1.37 $\times$ 10^-7^ |
| 3 | 3 | 4 | 4.47 $\times$ 10^-5^ | 8.98 $\times$ 10^-3^ | 4.01 $\times$ 10^-7^ | 1 | 3 | 4 | -4.22 $\times$ 10^-4^ | 3.15 $\times$ 10^-4^ | -1.33 $\times$ 10^-7^ |
| 3 | 3 | 3 | 2.52 $\times$ 10^-6^ | 1.04 $\times$ 10^-1^ | 2.62 $\times$ 10^-7^ | 3 | 4 | 1 | -4.22 $\times$ 10^-4^ | 3.12 $\times$ 10^-4^ | -1.32 $\times$ 10^-7^ |

| -1, 100 mM NaCl, 6 mM MgCl_2_ | | | | | | | | | | | |
| --- | --- | --- | --- | --- | --- | --- | --- | --- | --- | --- | --- |
| Positive terms | | | | | | Negative terms | | | | | |
| $i$ | $j$ | $k$ | $\delta\bar{v}_{i}\delta\bar{v}_{j}\delta\bar{v}_{k}$ | $w_{ijk}$ | $w_{ijk}\delta\bar{v}_{i}\delta\bar{v}_{j}\delta\bar{v}_{k}$ | $i$ | $j$ | $k$ | $\delta\bar{v}_{i}\delta\bar{v}_{j}\delta\bar{v}_{k}$ | $w_{ijk}$ | $w_{ijk}\delta\bar{v}_{i}\delta\bar{v}_{j}\delta\bar{v}_{k}$ |
| 4 | 4 | 4 | 6.39 $\times$ 10^-3^ | 2.17 $\times$ 10^-4^ | 1.38 $\times$ 10^-6^ | 2 | 2 | 2 | -2.52 $\times$ 10^-6^ | 1.41 $\times$ 10^-1^ | -3.56 $\times$ 10^-7^ |
| 3 | 3 | 3 | 4.67 $\times$ 10^-7^ | 6.50 $\times$ 10^-1^ | 3.04 $\times$ 10^-7^ | 3 | 3 | 1 | -6.89 $\times$ 10^-6^ | 1.80 $\times$ 10^-2^ | -1.24 $\times$ 10^-7^ |
| 3 | 1 | 2 | 1.21 $\times$ 10^-5^ | 4.66 $\times$ 10^-3^ | 5.64 $\times$ 10^-8^ | 1 | 3 | 3 | -6.89 $\times$ 10^-6^ | 1.76 $\times$ 10^-2^ | -1.21 $\times$ 10^-7^ |
| 2 | 1 | 3 | 1.21 $\times$ 10^-5^ | 4.67 $\times$ 10^-3^ | 5.16 $\times$ 10^-8^ | 1 | 2 | 2 | -2.12 $\times$ 10^-5^ | 5.02 $\times$ 10^-3^ | -1.06 $\times$ 10^-7^ |
| 3 | 1 | 1 | 1.02 $\times$ 10^-4^ | 5.04 $\times$ 10^-4^ | 5.13 $\times$ 10^-8^ | 2 | 2 | 1 | -2.12 $\times$ 10^-5^ | 4.69 $\times$ 10^-3^ | -9.94 $\times$ 10^-8^ |
| 1 | 1 | 3 | 1.02 $\times$ 10^-4^ | 4.93 $\times$ 10^-4^ | 5.02 $\times$ 10^-8^ | 3 | 1 | 3 | -6.89 $\times$ 10^-6^ | 1.42 $\times$ 10^-2^ | -9.80 $\times$ 10^-8^ |
| 1 | 3 | 1 | 1.02 $\times$ 10^-4^ | 4.86 $\times$ 10^-4^ | 4.95 $\times$ 10^-8^ | 1 | 2 | 1 | -1.78 $\times$ 10^-4^ | 1.67 $\times$ 10^-4^ | -2.97 $\times$ 10^-8^ |
| 2 | 2 | 3 | 1.44 $\times$ 10^-6^ | 2.93 $\times$ 10^-2^ | 4.21 $\times$ 10^-8^ | 2 | 1 | 2 | -2.12 $\times$ 10^-5^ | 1.40 $\times$ 10^-3^ | -2.97 $\times$ 10^-8^ |

| -2, 300 mM NaCl, 6 mM MgCl_2_ | | | | | | | | | | | |
| --- | --- | --- | --- | --- | --- | --- | --- | --- | --- | --- | --- |
| Positive terms | | | | | | Negative terms | | | | | |
| $i$ | $j$ | $k$ | $\delta\bar{v}_{i}\delta\bar{v}_{j}\delta\bar{v}_{k}$ | $w_{ijk}$ | $w_{ijk}\delta\bar{v}_{i}\delta\bar{v}_{j}\delta\bar{v}_{k}$ | $i$ | $j$ | $k$ | $\delta\bar{v}_{i}\delta\bar{v}_{j}\delta\bar{v}_{k}$ | $w_{ijk}$ | $w_{ijk}\delta\bar{v}_{i}\delta\bar{v}_{j}\delta\bar{v}_{k}$ |
| 3 | 3 | 3 | 7.26 $\times$ 10^-5^ | 2.79 $\times$ 10^-2^ | 2.02 $\times$ 10^-6^ | 1 | 1 | 1 | -4.11 $\times$ 10^-4^ | 9.08 $\times$ 10^-4^ | -3.73 $\times$ 10^-7^ |
| 4 | 4 | 4 | 6.37 $\times$ 10^-3^ | 3.06 $\times$ 10^-4^ | 1.95 $\times$ 10^-6^ | 1 | 2 | 2 | -2.02 $\times$ 10^-6^ | 7.75 $\times$ 10^-2^ | -1.56 $\times$ 10^-7^ |
| 1 | 2 | 1 | 2.88 $\times$ 10^-5^ | 8.39 $\times$ 10^-3^ | 2.42 $\times$ 10^-7^ | 2 | 2 | 1 | -2.02 $\times$ 10^-6^ | 7.75 $\times$ 10^-2^ | -1.56 $\times$ 10^-7^ |
| 1 | 1 | 2 | 2.88 $\times$ 10^-5^ | 8.38 $\times$ 10^-3^ | 2.42 $\times$ 10^-7^ | 2 | 1 | 2 | -2.02 $\times$ 10^-6^ | 7.74 $\times$ 10^-2^ | -1.56 $\times$ 10^-7^ |
| 2 | 1 | 1 | 2.88 $\times$ 10^-5^ | 8.38 $\times$ 10^-3^ | 2.42 $\times$ 10^-7^ | 4 | 4 | 1 | -2.56 $\times$ 10^-3^ | 5.46 $\times$ 10^-5^ | -1.40 $\times$ 10^-7^ |
| 2 | 2 | 2 | 1.42 $\times$ 10^-7^ | 7.16 $\times$ 10^-1^ | 1.01 $\times$ 10^-7^ | 1 | 4 | 4 | -2.56 $\times$ 10^-3^ | 5.46 $\times$ 10^-5^ | -1.40 $\times$ 10^-7^ |
| 4 | 4 | 2 | 1.79 $\times$ 10^-4^ | 4.06 $\times$ 10^-4^ | 7.28 $\times$ 10^-8^ | 4 | 1 | 2 | -7.18 $\times$ 10^-5^ | 1.23 $\times$ 10^-4^ | -8.86 $\times$ 10^-9^ |
| 2 | 4 | 4 | 1.79 $\times$ 10^-4^ | 4.06 $\times$ 10^-4^ | 7.28 $\times$ 10^-8^ | 2 | 1 | 4 | -7.18 $\times$ 10^-5^ | 1.23 $\times$ 10^-4^ | -8.86 $\times$ 10^-9^ |

| -2, 100 mM NaCl, 6 mM MgCl_2_ | | | | | | | | | | | |
| --- | --- | --- | --- | --- | --- | --- | --- | --- | --- | --- | --- |
| Positive terms | | | | | | Negative terms | | | | | |
| $i$ | $j$ | $k$ | $\delta\bar{v}_{i}\delta\bar{v}_{j}\delta\bar{v}_{k}$ | $w_{ijk}$ | $w_{ijk}\delta\bar{v}_{i}\delta\bar{v}_{j}\delta\bar{v}_{k}$ | $i$ | $j$ | $k$ | $\delta\bar{v}_{i}\delta\bar{v}_{j}\delta\bar{v}_{k}$ | $w_{ijk}$ | $w_{ijk}\delta\bar{v}_{i}\delta\bar{v}_{j}\delta\bar{v}_{k}$ |
| 4 | 4 | 4 | 5.26 $\times$ 10^-3^ | 4.21 $\times$ 10^-4^ | 2.21 $\times$ 10^-6^ | 2 | 2 | 2 | -2.80 $\times$ 10^-5^ | 8.77 $\times$ 10^-2^ | -2.46 $\times$ 10^-6^ |
| 2 | 2 | 4 | 1.60 $\times$ 10^-4^ | 1.37 $\times$ 10^-2^ | 2.20 $\times$ 10^-6^ | 2 | 4 | 4 | -9.18 $\times$ 10^-4^ | 2.39 $\times$ 10^-3^ | -2.19 $\times$ 10^-6^ |
| 4 | 2 | 2 | 1.60 $\times$ 10^-4^ | 1.37 $\times$ 10^-2^ | 2.20 $\times$ 10^-6^ | 4 | 4 | 2 | -9.18 $\times$ 10^-4^ | 2.39 $\times$ 10^-3^ | -2.19 $\times$ 10^-6^ |
| 2 | 4 | 2 | 1.60 $\times$ 10^-4^ | 1.36 $\times$ 10^-2^ | 2.18 $\times$ 10^-6^ | 4 | 2 | 4 | -9.18 $\times$ 10^-4^ | 2.14 $\times$ 10^-3^ | -1.97 $\times$ 10^-6^ |
| 4 | 1 | 2 | 4.04 $\times$ 10^-4^ | 3.97 $\times$ 10^-3^ | 1.61 $\times$ 10^-6^ | 1 | 2 | 2 | -7.06 $\times$ 10^-5^ | 2.30 $\times$ 10^-2^ | -1.63 $\times$ 10^-6^ |
| 2 | 1 | 4 | 4.04 $\times$ 10^-4^ | 3.97 $\times$ 10^-3^ | 1.61 $\times$ 10^-6^ | 2 | 2 | 1 | -7.06 $\times$ 10^-5^ | 2.30 $\times$ 10^-2^ | -1.63 $\times$ 10^-6^ |
| 2 | 4 | 1 | 4.04 $\times$ 10^-4^ | 3.97 $\times$ 10^-3^ | 1.60 $\times$ 10^-6^ | 4 | 4 | 1 | -2.32 $\times$ 10^-3^ | 6.98 $\times$ 10^-4^ | -1.62 $\times$ 10^-6^ |
| 1 | 4 | 2 | 4.04 $\times$ 10^-4^ | 3.97 $\times$ 10^-3^ | 1.60 $\times$ 10^-6^ | 1 | 4 | 4 | -2.32 $\times$ 10^-3^ | 6.98 $\times$ 10^-4^ | -1.62 $\times$ 10^-6^ |

| -2, 20 mM NaCl, 6 mM MgCl_2_ | | | | | | | | | | | |
| --- | --- | --- | --- | --- | --- | --- | --- | --- | --- | --- | --- |
| Positive terms | | | | | | Negative terms | | | | | |
| $i$ | $j$ | $k$ | $\delta\bar{v}_{i}\delta\bar{v}_{j}\delta\bar{v}_{k}$ | $w_{ijk}$ | $w_{ijk}\delta\bar{v}_{i}\delta\bar{v}_{j}\delta\bar{v}_{k}$ | $i$ | $j$ | $k$ | $\delta\bar{v}_{i}\delta\bar{v}_{j}\delta\bar{v}_{k}$ | $w_{ijk}$ | $w_{ijk}\delta\bar{v}_{i}\delta\bar{v}_{j}\delta\bar{v}_{k}$ |
| 4 | 4 | 4 | 6.13 $\times$ 10^-3^ | 9.54 $\times$ 10^-4^ | 5.85 $\times$ 10^-6^ | 4 | 4 | 1 | -3.90 $\times$ 10^-3^ | 6.69 $\times$ 10^-5^ | -2.61 $\times$ 10^-7^ |
| 3 | 3 | 3 | 1.34 $\times$ 10^-4^ | 4.17 $\times$ 10^-2^ | 5.59 $\times$ 10^-6^ | 1 | 4 | 4 | -3.90 $\times$ 10^-3^ | 6.69 $\times$ 10^-5^ | -2.61 $\times$ 10^-7^ |
| 4 | 4 | 2 | 5.58 $\times$ 10^-5^ | 7.83 $\times$ 10^-4^ | 4.37 $\times$ 10^-8^ | 1 | 1 | 1 | -1.58 $\times$ 10^-3^ | 6.56 $\times$ 10^-5^ | -1.04 $\times$ 10^-7^ |
| 2 | 4 | 4 | 5.58 $\times$ 10^-5^ | 7.82 $\times$ 10^-4^ | 4.36 $\times$ 10^-8^ | 3 | 3 | 1 | -3.05 $\times$ 10^-4^ | 8.68 $\times$ 10^-5^ | -2.65 $\times$ 10^-8^ |
| 1 | 2 | 1 | 2.26 $\times$ 10^-5^ | 1.53 $\times$ 10^-3^ | 3.46 $\times$ 10^-8^ | 1 | 3 | 3 | -3.05 $\times$ 10^-4^ | 4.11 $\times$ 10^-5^ | -1.25 $\times$ 10^-8^ |
| 1 | 1 | 2 | 2.26 $\times$ 10^-5^ | 1.53 $\times$ 10^-3^ | 3.46 $\times$ 10^-8^ | 1 | 2 | 2 | -3.23 $\times$ 10^-7^ | 3.58 $\times$ 10^-2^ | -1.16 $\times$ 10^-8^ |
| 2 | 1 | 1 | 2.26 $\times$ 10^-5^ | 1.53 $\times$ 10^-3^ | 3.45 $\times$ 10^-8^ | 2 | 2 | 1 | -3.23 $\times$ 10^-7^ | 3.58 $\times$ 10^-2^ | -1.16 $\times$ 10^-8^ |
| 4 | 1 | 1 | 2.48 $\times$ 10^-3^ | 5.17 $\times$ 10^-6^ | 1.28 $\times$ 10^-8^ | 2 | 1 | 2 | -3.23 $\times$ 10^-7^ | 3.58 $\times$ 10^-2^ | -1.15 $\times$ 10^-8^ |

| -2, 20 mM NaCl, 0 mM MgCl_2_ | | | | | | | | | | | |
| --- | --- | --- | --- | --- | --- | --- | --- | --- | --- | --- | --- |
| Positive terms | | | | | | Negative terms | | | | | |
| $i$ | $j$ | $k$ | $\delta\bar{v}_{i}\delta\bar{v}_{j}\delta\bar{v}_{k}$ | $w_{ijk}$ | $w_{ijk}\delta\bar{v}_{i}\delta\bar{v}_{j}\delta\bar{v}_{k}$ | $i$ | $j$ | $k$ | $\delta\bar{v}_{i}\delta\bar{v}_{j}\delta\bar{v}_{k}$ | $w_{ijk}$ | $w_{ijk}\delta\bar{v}_{i}\delta\bar{v}_{j}\delta\bar{v}_{k}$ |
| 4 | 4 | 4 | 1.39 $\times$ 10^-3^ | 1.34 $\times$ 10^-2^ | 1.86 $\times$ 10^-5^ | 4 | 4 | 1 | -9.88 $\times$ 10^-4^ | 1.34 $\times$ 10^-2^ | -1.32 $\times$ 10^-5^ |
| 1 | 4 | 1 | 7.03 $\times$ 10^-4^ | 1.34 $\times$ 10^-2^ | 9.42 $\times$ 10^-6^ | 1 | 4 | 4 | -9.88 $\times$ 10^-4^ | 1.34 $\times$ 10^-2^ | -1.32 $\times$ 10^-5^ |
| 4 | 1 | 1 | 7.03 $\times$ 10^-4^ | 1.34 $\times$ 10^-2^ | 9.42 $\times$ 10^-6^ | 4 | 1 | 4 | -9.88 $\times$ 10^-4^ | 1.34 $\times$ 10^-2^ | -1.32 $\times$ 10^-5^ |
| 1 | 1 | 4 | 7.03 $\times$ 10^-4^ | 1.34 $\times$ 10^-2^ | 9.42 $\times$ 10^-6^ | 1 | 1 | 1 | -5.00 $\times$ 10^-4^ | 1.34 $\times$ 10^-2^ | -6.70 $\times$ 10^-6^ |
| 5 | 5 | 5 | 5.96 $\times$ 10^-3^ | 5.56 $\times$ 10^-4^ | 3.31 $\times$ 10^-6^ | 2 | 4 | 4 | -2.51 $\times$ 10^-4^ | 1.03 $\times$ 10^-2^ | -2.58 $\times$ 10^-6^ |
| 3 | 4 | 4 | 6.57 $\times$ 10^-4^ | 3.59 $\times$ 10^-3^ | 2.36 $\times$ 10^-6^ | 4 | 4 | 2 | -2.51 $\times$ 10^-4^ | 1.03 $\times$ 10^-2^ | -2.58 $\times$ 10^-6^ |
| 4 | 4 | 3 | 6.57 $\times$ 10^-4^ | 3.59 $\times$ 10^-3^ | 2.36 $\times$ 10^-6^ | 2 | 2 | 2 | -8.15 $\times$ 10^-6^ | 3.14 $\times$ 10^-1^ | -2.56 $\times$ 10^-6^ |
| 2 | 1 | 4 | 1.78 $\times$ 10^-4^ | 1.03 $\times$ 10^-2^ | 1.84 $\times$ 10^-6^ | 3 | 1 | 4 | -4.67 $\times$ 10^-4^ | 3.59 $\times$ 10^-3^ | -1.68 $\times$ 10^-6^ |
