# Supplementary material for: Site-specific free energy surface parameters from single-molecule fluorescence measurements of exciton-coupled (iCy3)2 dimer probes positioned at DNA replication fork junctions
Source: Nucleic Acids Res. 2025 Feb 5;53(3):gkaf047. doi: 10.1093/nar/gkaf047 (PMC11795206; doi:10.1093/nar/gkaf047)
Supplement: gkaf047_Supplemental_Files [file gkaf047_supplemental_files.zip › Supplementary Tables.zip!/TableS5.docx]

**Table S5. Optimized forward and backward inverse rate constants corresponding to the elementary reactive steps for the samples studied.**

| Sample | $k_{12}^{-1}$ | $k_{21}^{-1}$ | $k_{13}^{-1}$ | $k_{31}^{-1}$ | $k_{14}^{-1}$ | $k_{41}^{-1}$ | $k_{23}^{-1}$ | $k_{32}^{-1}$ | $k_{24}^{-1}$ | $k_{42}^{-1}$ | $k_{34}^{-1}$ | $k_{43}^{-1}$ | $k_{15}^{-1}$ | $k_{51}^{-1}$ | $k_{25}^{-1}$ | $k_{52}^{-1}$ | $k_{35}^{-1}$ | $k_{53}^{-1}$ | $k_{45}^{-1}$ | $k_{54}^{-1}$ |
| --- | --- | --- | --- | --- | --- | --- | --- | --- | --- | --- | --- | --- | --- | --- | --- | --- | --- | --- | --- | --- |
| +1, 300 mM NaCl | 66.3 | 533 | - | - | - | - | - | - | - | - | 11.8e4 | 363 | 0.85 | 1.75 | - | - | 556 | 9.91 | - | - |
| +1, 100 mM NaCl | - | - | - | - | 4.97 | 4.86 | 66.1e3 | 548 | 17.7e2 | 95.4 | 1.84 | 0.28 | - | - | - | - | - | - | - | - |
| +1, 20 mM NaCl | 0.55 | 1.64 | - | - | 16.4e2 | 15.4 | 27.1e3 | 627 | - | - | - | - | - | - | - | - | - | - | - | - |
| +1, 20 mM NaCl, 0 mM MgCl_2_ | - | - | 4.64 | 121 | - | - | 25.1e2 | 109 | 731 | 368 | 4.31 | 0.37 | - | - | - | - | - | - | - | - |
| -1, 100 mM NaCl | 4.27 | 39.0 | 1.45 | 41.2 | 77.6e2 | 82.7 | 433 | 11.2e4 | - | - | - | - | - | - | - | - | - | - | - | - |
| -2, 300 mM NaCl | 0.40 | 3.74 | - | - | 539 | 10.8 | 21.8e3 | 518 | - | - | - | - | - | - | - | - | - | - | - | - |
| -2, 100 mM NaCl | 2.20 | 8.32 | - | - | 0.71 | 0.42 | 109 | 382 | 35.0e3 | 78.6e3 | - | - | - | - | - | - | - | - | - | - |
| -2, 20 mM NaCl | 0.55 | 12.9 | 52.5e3 | 494 | 180 | 15.6 | 84.5e2 | 18.6e2 | - | - | - | - | - | - | - | - | - | - | - | - |
| -2, 20 mM NaCl, 0 mM MgCl_2_ | - | - | 10.4 | 12.7 | 0.0005 | 0.0005 | 4.65 | 1.17 | - | - | 75.0e3 | 91.7e3 | 87.2e3 | 285 | - | - | - | - | - | - |

Inverse rate constants defined in Eq. (4), listed in units of milliseconds. Error bars are < 1% of reported values (see Statistical Uncertainty Analysis below).
