# Supplementary material for: Site-specific free energy surface parameters from single-molecule fluorescence measurements of exciton-coupled (iCy3)2 dimer probes positioned at DNA replication fork junctions
Source: Nucleic Acids Res. 2025 Feb 5;53(3):gkaf047. doi: 10.1093/nar/gkaf047 (PMC11795206; doi:10.1093/nar/gkaf047)
Supplement: gkaf047_Supplemental_Files [file gkaf047_supplemental_files.zip › Supplementary Tables.zip!/TableS6.docx]

**Table S6. Relative free energy minima.**

| Sample | $G_{1}$ | $G_{2}$ | $G_{3}$ | $G_{4}$ | $G_{5}$ |
| --- | --- | --- | --- | --- | --- |
| +1, 300 mM NaCl | 3.8 | 2.9 | 0 | 5.7 | 3.8 |
| +1, 100 mM NaCl | 3.7 | 0 | 1.8 | 3.7 | - |
| +1, 20 mM NaCl | 1.1 | 0 | 3.6 | 5.9 | - |
| +1, 20 mM NaCl, 0 mM MgCl_2_ | 4.4 | 0 | 1.6 | 4.4 | - |
| -1, 100 mM NaCl | 3.6 | 1.2 | 0 | 5.5 | - |
| -2, 300 mM NaCl | 2.2 | 0 | 3.8 | 6.8 | - |
| -2, 100 mM NaCl | 2.5 | 1.3 | 0 | 3.2 | - |
| -2, 20 mM NaCl | 3.1 | 0 | 3.1 | 5.7 | - |
| -2, 20 mM NaCl, 0 mM MgCl_2_ | 1.6 | 0 | 1.4 | 1.6 | 6.4 |

The ground state is taken to be the macrostate with minimum free energy, $G_{min}$, and excited state energies are given by $G_{i}=-k_{B}T\ln\left( {p_{i}^{eq}}/{\sigma_{i}\sqrt{2\pi}} \right)-G_{min}$ with parameters $\sigma_{i}$ and $p_{i}^{eq}$ listed in Table S1 and Table S2, respectively. Energies are listed in units of $k_{B}T$.
