# Supplementary material for: Site-specific free energy surface parameters from single-molecule fluorescence measurements of exciton-coupled (iCy3)2 dimer probes positioned at DNA replication fork junctions
Source: Nucleic Acids Res. 2025 Feb 5;53(3):gkaf047. doi: 10.1093/nar/gkaf047 (PMC11795206; doi:10.1093/nar/gkaf047)
Supplement: gkaf047_Supplemental_Files [file gkaf047_supplemental_files.zip › Supplementary Tables.zip!/TableS7.docx]

**Table S7. Free energies of activation corresponding to the elementary reactive steps.**

| Sample | $G_{12}^{\ddagger}$ | $G_{21}^{\ddagger}$ | $G_{13}^{\ddagger}$ | $G_{31}^{\ddagger}$ | $G_{14}^{\ddagger}$ | $G_{41}^{\ddagger}$ | $G_{23}^{\ddagger}$ | $G_{32}^{\ddagger}$ | $G_{24}^{\ddagger}$ | $G_{42}^{\ddagger}$ | $G_{34}^{\ddagger}$ | $G_{43}^{\ddagger}$ | $G_{15}^{\ddagger}$ | $G_{51}^{\ddagger}$ | $G_{25}^{\ddagger}$ | $G_{52}^{\ddagger}$ | $G_{35}^{\ddagger}$ | $G_{53}^{\ddagger}$ | $G_{45}^{\ddagger}$ | $G_{54}^{\ddagger}$ |
| --- | --- | --- | --- | --- | --- | --- | --- | --- | --- | --- | --- | --- | --- | --- | --- | --- | --- | --- | --- | --- |
| +1, 300 mM NaCl | 4.4 | 6.4 | - | - | - | - | - | - | - | - | 11.8 | 6.1 | 0 | 0.7 | - | - | 6.5 | 6.2 | - | - |
| +1, 100 mM NaCl | - | - | - | - | 2.9 | 2.9 | 12.4 | 7.6 | 8.8 | 1.9 | 1.9 | 0 | - | - | - | - | - | - | - | - |
| +1, 20 mM NaCl | 0 | 1.1 | - | - | 8.0 | 3.2 | 10.8 | 7.0 | - | - | - | - | - | - | - | - | - | - | - | - |
| +1, 20 mM NaCl, 0 mM MgCl_2_ | - | - | 2.5 | 5.8 | - | - | 8.8 | 5.7 | 7.6 | 6.9 | 2.4 | 0 | - | - | - | - | - | - | - | - |
| -1, 100 mM NaCl | 1.1 | 3.3 | 0 | 3.3 | 8.6 | 4.0 | 5.7 | 11.2 | - | - | - | - | - | - | - | - | - | - | - | - |
| -2, 300 mM NaCl | 0 | 2.2 | - | - | 7.2 | 3.3 | 10.9 | 7.1 | - | - | - | - | - | - | - | - | - | - | - | - |
| -2, 100 mM NaCl | 1.7 | 3.0 | - | - | 0.5 | 0 | 5.5 | 6.8 | - | - | - | - | - | - | - | - | - | - | - | - |
| -2, 20 mM NaCl | 0 | 3.2 | 11.5 | 6.8 | 5.8 | 3.3 | 9.6 | 8.1 | - | - | - | - | - | - | - | - | - | - | - | - |
| -2, 20 mM NaCl, 0 mM MgCl_2_ | - | - | 9.9 | 10.1 | 0 | 0 | 9.1 | 7.8 | - | - | 18.8 | 19.0 | 19.0 | 13.2 | - | - | - | - | - | - |

The activation energies are determined from $G_{ij}^{\ddagger}=-k_{B}T\ln\left( {k_{ij}}/{k_{max}} \right)$ where $k_{max}$ is the fastest rate constant determined from the analysis. Energies are listed in units of $k_{B}T$.
