# Supplementary material for: Site-specific free energy surface parameters from single-molecule fluorescence measurements of exciton-coupled (iCy3)2 dimer probes positioned at DNA replication fork junctions
Source: Nucleic Acids Res. 2025 Feb 5;53(3):gkaf047. doi: 10.1093/nar/gkaf047 (PMC11795206; doi:10.1093/nar/gkaf047)
Supplement: gkaf047_Supplemental_Files [file gkaf047_supplemental_files.zip › Supplementary Tables.zip!/TableS8.docx]

**Table S8. Free energies of activation corresponding to the elementary reactive steps.**

| Sample | $G_{12}^{o\ddagger}$ | $G_{21}^{o\ddagger}$ | $G_{13}^{o\ddagger}$ | $G_{31}^{o\ddagger}$ | $G_{14}^{o\ddagger}$ | $G_{41}^{o\ddagger}$ | $G_{23}^{o\ddagger}$ | $G_{32}^{o\ddagger}$ | $G_{24}^{o\ddagger}$ | $G_{42}^{o\ddagger}$ | $G_{34}^{o\ddagger}$ | $G_{43}^{o\ddagger}$ | $G_{15}^{o\ddagger}$ | $G_{51}^{o\ddagger}$ | $G_{25}^{o\ddagger}$ | $G_{52}^{o\ddagger}$ | $G_{35}^{o\ddagger}$ | $G_{53}^{o\ddagger}$ | $G_{45}^{o\ddagger}$ | $G_{54}^{o\ddagger}$ |
| --- | --- | --- | --- | --- | --- | --- | --- | --- | --- | --- | --- | --- | --- | --- | --- | --- | --- | --- | --- | --- |
| +1, 300 mM NaCl | 8.2 | 9.3 | - | - | - | - | - | - | - | - | 11.8 | 11.8 | 3.8 | 4.5 | - | - | 6.5 | 6.2 | - | - |
| +1, 100 mM NaCl | - | - | - | - | 6.6 | 6.6 | 12.4 | 9.4 | 8.8 | 9.5 | 3.7 | 3.7 | - | - | - | - | - | - | - | - |
| +1, 20 mM NaCl | 1.1 | 1.1 | - | - | 9.1 | 9.1 | 10.8 | 10.6 | - | - | - | - | - | - | - | - | - | - | - | - |
| +1, 20 mM NaCl, 0 mM MgCl_2_ | - | - | 6.9 | 7.4 | - | - | 8.8 | 7.3 | 7.6 | 11.3 | 4.0 | 4.4 | - | - | - | - | - | - | - | - |
| -1, 100 mM NaCl | 4.7 | 4.5 | 3.6 | 3.3 | 12.2 | 9.5 | 6.9 | 11.2 | - | - | - | - | - | - | - | - | - | - | - | - |
| -2, 300 mM NaCl | 2.2 | 2.2 | - | - | 9.4 | 10.1 | 10.9 | 10.9 | - | - | - | - | - | - | - | - | - | - | - | - |
| -2, 100 mM NaCl | 4.2 | 4.3 | - | - | 3.0 | 3.2 | 6.8 | 6.8 | - | - | - | - | - | - | - | - | - | - | - | - |
| -2, 20 mM NaCl | 3.1 | 3.2 | 14.6 | 9.9 | 8.9 | 9.0 | 9.6 | 11.2 | - | - | - | - | - | - | - | - | - | - | - | - |
| -2, 20 mM NaCl, 0 mM MgCl_2_ | - | - | 11.5 | 11.5 | 1.6 | 1.6 | 9.1 | 9.2 | - | - | 20.2 | 20.6 | 20.6 | 19.6 | - | - | - | - | - | - |

The activation energies are determined from $G_{ij}^{0\ddagger}=-k_{B}T\ln\left( {k_{ij}}/{k_{max}} \right)+G_{i}$ where $k_{max}$ is the fastest rate constant determined from the analysis and $G_{i}$ is the free energy minimum of the *i*th macrostate. Energies are listed in units of $k_{B}T$.
